# Association studies of WD repeat domain 3 and chitobiosyldiphosphodolichol beta-mannosyltransferase genes with schizophrenia in a Japanese population

**DOI:** 10.1371/journal.pone.0190991

**Published:** 2018-01-08

**Authors:** Momoko Kobayashi, Daisuke Jitoku, Yoshimi Iwayama, Naoki Yamamoto, Tomoko Toyota, Katsuaki Suzuki, Mitsuru Kikuchi, Tasuku Hashimoto, Nobuhisa Kanahara, Akeo Kurumaji, Takeo Yoshikawa, Toru Nishikawa

**Affiliations:** 1 Department of Psychiatry and Behavioral Sciences, Graduate School of Tokyo Medical and Dental University, Bunkyo-ku, Tokyo, Japan; 2 Laboratory for Molecular Psychiatry, RIKEN Brain Science Institute, Wako, Saitama, Japan; 3 Department of Psychiatry, Hamamatsu University School of Medicine, Hamamatsu, Shizuoka, Japan; 4 Department of Psychiatry and Neurobiology, Graduate School of Medical Science, Kanazawa University, Kanazawa, Ishikawa, Japan; 5 Department of Psychiatry, Graduate School of Medicine, Chiba University, Chiba, Chiba, Japan; Peking University, Institute of Mental Health, CHINA

## Abstract

Schizophrenia and schizophrenia-like symptoms induced by the dopamine agonists and *N*-methyl-D aspartate type glutamate receptor antagonists occur only after the adolescent period. Similarly, animal models of schizophrenia by these drugs are also induced after the critical period around postnatal week three. Based upon the development-dependent onsets of these psychotomimetic effects, by using a DNA microarray technique, we identified the WD repeat domain 3 (*WDR3*) and chitobiosyldiphosphodolichol beta-mannosyltransferase (*ALG1*) genes as novel candidates for schizophrenia-related molecules, whose mRNAs were up-regulated in the adult (postnatal week seven), but not in the infant (postnatal week one) rats by an indirect dopamine agonist, and phencyclidine, an antagonist of the NMDA receptor. WDR3 and other related proteins are the nuclear proteins presumably involved in various cellular activities, such as cell cycle progression, signal transduction, apoptosis, and gene regulation. ALG1 is presumed to be involved in the regulation of the protein *N*-glycosylation. To further elucidate the molecular pathophysiology of schizophrenia, we have evaluated the genetic association of *WDR3* and *ALG1* in schizophrenia. We examined 21 single nucleotide polymorphisms [SNPs; W1 (rs1812607)-W16 (rs6656360), A1 (rs8053916)-A10 (rs9673733)] from these genes using the Japanese case-control sample (1,808 schizophrenics and 2,170 matched controls). No significant genetic associations of these SNPs were identified. However, we detected a significant association of W4 (rs319471) in the female schizophrenics (allelic *P* = 0.003, genotypic *P* = 0.008). Based on a haplotype analysis, the observed haplotypes consisting of W4 (rs319471)–W5 (rs379058) also displayed a significant association in the female schizophrenics (*P* = 0.016). Even after correction for multiple testing, these associations remained significant. Our findings suggest that the *WDR3* gene may likely be a sensitive factor in female patients with schizophrenia, and that modification of the WDR3 signaling pathway warrants further investigation as to the pathophysiology of schizophrenia.

## Introduction

Schizophrenia typically develops after adolescence [1]. Methamphetamine, an indirect dopamine agonist, and phencyclidine (PCP) and ketamine, antagonists of the *N*-methyl-D aspartate (NMDA) type glutamate receptor, are known to cause schizophrenia-like symptoms only after the adolescent period [2–4]. Similarly, in experimental animals, the psychotomimetic effects of these drugs have also been observed after the critical period around postnatal week three [5–7]. These observations suggest that the neuron circuits and molecules in the brain related to schizophrenia might show an age-related response to these psychotomimetics. In support of this assumption, we have found that methamphetamine and PCP elicit developmental changes in the c-Fos protein expression pattern, which reflects activity modification of the cell activities in the nervous systems, in the rat neocortex across the critical period [8, 9]. Consequently, we have explored the gene transcripts that are developmentally regulated after methamphetamine and PCP administrations in the rat cerebral neocortex. Based on this series of experiments using a DNA microarray technique, we detected as candidates for this type of novel schizophrenia-related genes the WD repeat domain 3 (*WDR3*) and chitobiosyldiphosphodolichol beta-mannosyltransferase (*ALG1*), whose mRNAs were up-regulated greater in the adult (postnatal days 50) than in the infant (postnatal days 8) rats by these schizophrenomimetics. Furthermore, these genes are located in linkage regions with schizophrenia [10, 11]. *WDR3*, also known as DIP2 and UTP12, is broadly expressed including in the brain [12]. This protein is contained in the nuclear, nucleolus and the main component of the small 40S ribosome subunit [12–14]. It also plays an essential role in the processing of 18S rRNA [14]. However, the specific function in the brain is unexplained. On the one hand, ALG1 is presumed to be involved in the regulation of the protein *N*-glycosylation, having the activity to add the first mannose residue to the lipid-linked oligosaccharides [15]. Abnormal glycosylation of the glutamate transporter, which may also be regulated by *N*-glycosylation, was reported in the postmortem study of schizophrenia [16]. Therefore, *WDR3* and *ALG1* may also be associated with the susceptibility and/or pathogenesis of schizophrenia.

In the present study, we used single nucleotide polymorphisms (SNPs) in the Japanese case-control sample to implement a genetic association study of the *WDR3* and *ALG1* genes in schizophrenia.

## Materials and methods

### Subjects

We analyzed 1,808 schizophrenics (male N = 992; mean age 48.9 ± 13.7 years, female N = 816; mean age 50.9 ± 14.2 years) and 2,170 matched controls (male N = 889; mean age 39.2 ± 13.8 years, female N = 1,281; mean age 44.6 ± 14.1 years) from the Japanese population. All the case-control subjects were assembled from the Honshu area of Japan (the main island of the nation). The populations of Honshu are categorized as a single genetic cluster [17, 18]. For the same subset used in a previous study [18], the Pr (K = 1) value (specifically, number of population present in sample = 1 [19]) was greater than 0.99 [20, 21], and k (the genomic control factor [22]) was 1.074. These data showed a negligible population stratification effect in our Japanese samples [23]. All patients were diagnosed by well-trained psychiatrists based on the Diagnostic and Statistical Manual of Mental Disorders, fourth edition (DSM-IV Criteria). The control subjects were assembled from hospital staff and volunteers. Expert psychiatrists checked whether or not they have a present or past history of psychosis and a family history of mental disorder within the second degree of relationships by brief interviews. The present study was approved by the ethics committees of the Tokyo Medical and Dental University and RIKEN Brain Science Institute. All participants gave informed and written consent to participate in the study.

### Gene and SNP selection and genotyping

#### Exploration of target genes for association analysis

Before the gene and SNP selection and genotyping, we prepared the male Wistar rats (ST strain, Clea Japan, Japan) to explore of the novel candidate genes of schizophrenia. In this study, only male rats were used in the pharmacological experiment in order to avoid changes in behavior and biochemical response to various drugs due to the onset of the female menstrual cycle. The animals were bred under a 12 hour light / dark cycle (lights on 08:00 hours) at 24.0 ± 0.5 degrees (Celsius) and had free access to food and water. The animal experiments were approved by the ethics committee of animal experiment of the Tokyo Medical and Dental University, and were strictly performed following the guidelines of the university.

To explore the novel target genes for the present association analysis, we performed a DNA microarray analysis using the GeneChip® Rat Gene 1.0 ST Array (Affymetrix, Santa Clara, CA, USA) to find in the neocortex the developmentally regulated transcripts responsive to the psychotomimetic doses (adult period) of PCP and methamphetamine across the critical period around postnatal week three. The array system interrogates 27,342 well-annotated genes with 722,254 distinct probes. A detailed explanation can be found at https://www.affymetrix.com/support/technical/datasheets/gene_1_0_st_datasheet.pdf. Data analyses have been achieved by the software, GeneSpring GX 11.0 (Agilent Technologies, Santa Clara, CA, USA).

For this screening stage, six experimental groups of rats were prepared; five saline-administered control rats at PD50; five PCP (7.5 mg/kg, s.c.)-injected rats at PD50; five methamphetamine (4.8 mg/kg, s.c.)-injected rats at PD50; five saline-administered control rats at PD8; five PCP (7.5 mg/kg, s.c.)-injected rats at PD8; and five methamphetamine (4.8 mg/kg, s.c.)-injected rats at PD8. Equal amounts of the total RNA individually isolated from the respective five animals per each treatment group were combined. The cDNA synthesis, cRNA labeling, hybridization and scanning were done according to the manufacturer’s instructions (Affymetrix).

Based upon the DNA microarray data, we finally chose *WDR3* and *ALG1* as the genes for the present human association study by screening the transcripts of their rat homologues that showed the development-dependent upregulation by PCP and methamphetamine injection with the log2 ratio for the PCP/control (saline) and methamphetamine/control of more than 0.263 (1.2 times the control value) at PD 50 and that less than 0.137 (1.1 times the control value) in the ratio at PD 8 [log2 ratio of the *WDR3*: PCP 0.595 at PD50 (151%), 0.058 at PD8 (104%), methamphetamine 0.571 at PD50 (149%), 0.009 at PD8 (101%); log2 ratio of the *ALG1*: PCP 0.273 at PD50 (121%), 0.128 at PD8 (109%), methamphetamine 0.265 at PD50 (120%), 0.047 at PD8 (103%)].

#### Selection of SNPs and genotyping

We first retrieved the region 10kb up- and down-stream of these genes that provided the correlation coefficient of r^2^>0.85 and minor allele frequency of MAF>0.10 from the public databases [dbSNP (build 149) of the National Center for Biotechnology (NCBI) (http://www.ncbi.nlm.nih.gov/projects/SNP/)]. We then used Carlson’s LD-Select algorithm to evaluate the selection of the SNPs [24]. Additionally, we added SNPs from the insulator regions (CTCF binding site) between the target and adjacent gene as an effective region using CTCFBSDB 2.0 (http://insulatordb.uthsc.edu/) [25].

SNP genotyping was performed by TaqMan SNP genotyping assays (Applied Biosystems, Foster City, CA, USA). We used an ABI PRISM 7900HT (Applied Biosystems) or C1000 Touch Thermal Cycler with a 384-Well Reaction Module (BIO-RAD, Hercules, CA, USA) for the Polymerase Chain Reaction (PCR), and we analyzed the fluorescent signals using the 7900HT Sequence Detection System and SDS v2.3 software (Applied Biosystems).

### Statistical analyses

Fisher’s exact test using the PLINK v1.07 program was used to calculate the Hardy-Weinberg equilibrium (HWE) and the count of the alleles and genotypes in the case-control samples for association (http://zzz.bwh.harvard.edu/plink/) [26]. We calculated the *P*-value of the false discovery rate (FDR) using the Benjamini-Hochberg procedure as a multiple testing for deriving the observed significance to correct.

For analysis of the linkage disequilibrium (LD) test to estimate the degree of LD, we used two LD parameters, i.e., the standardized disequilibrium coefficient (*D’*) and *r*^*2*^, calculated by Haploview v4.2 (http://www.broad.mit.edu/mpg/haploview/) [27]. We computed the standardized disequilibrium coefficient based on *D’* according to the method of Gabriel et al. (2002) [28]. We executed the haplotype correlated analysis for common haplotypes (frequency≧0.05), then we calculated the individual and global haplotypic *P*-values using UNPHASED 3.1.4 (http://www.mrc-bsu.cam.ac.uk/personal/frank/software/unphased/). The multiple testing was calculated by FDR.

Moreover, we undertook an association analysis between these genes and schizophrenia in a stratified manner according to gender and age-at-onset using Fisher’s exact test with the PLINK v1.07 program. In the age-at-onset analysis, we divided the group into two age-at-onset categories, a) <18 years old, or 18 years old and greater, and b) <16 years old, 16–25 years old, 26–35 years old, or 36 years old and greater. In schizophrenia, even if the disease has similar symptoms, the age-at-onset of the disease with different causes occasionally changes. Therefore, the latter analysis is important to eliminate the possibility of heterogeneity which is considered to be present in schizophrenia. Moreover, schizophrenia with an onset age below 18 is often classified as early-onset schizophrenia in biological and clinical studies [29].

Furthermore, we examined the interaction of these genes using the multifactor dimensionality reduction (MDR) analysis [30], available in the open-source software package (http://www.multifactordimensionalityreduction.org/). An MDR analysis was performed by the MDR 3.0.2 program, and the permutation analysis used MDRpt Version 1.0.2 beta 2 (1,000 runs) for the testing accuracy and cross-validation consistency [31]. We used the false discovery rate as a multiple testing for the chi-square *P*-value. Before the analysis, the specific SNPs were excluded to avoid any false evaluation, the SNP showed a low MAF (<0.05), and the SNPs displayed a high LD (r^2^>0.95).

The statistical power was calculated by the genetic power calculator (http://zzz.bwh.harvard.edu/gpc/cc2.html). The assumptive parameter is as follows: An additive model with the genotypic relative risk = 1.3, prevalence of disease = 0.01, risk allele frequency = 0.2, type I error rate = 0.05 and 1-type II error rate (determine N) = 0.8. These tests were used to the level such that the statistical significance was set at *P*<0.05.

## Results

### Association result

**Fig 1:**
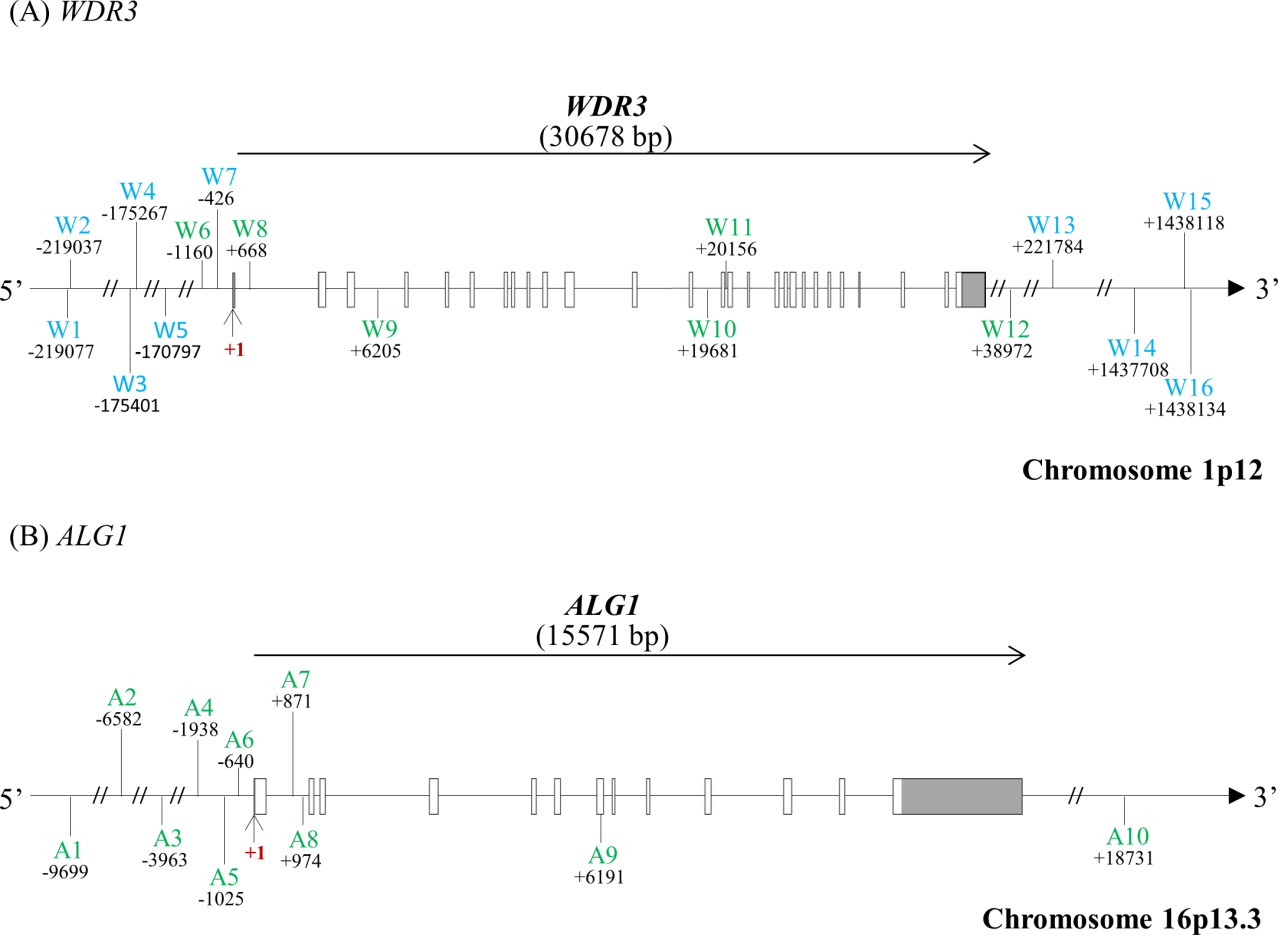
Genomic structure of human *WDR3* and *ALG1*. Genomic structures and positions of the SNPs in human *WDR3* (A) and *ALG1* (B). Exons are denoted by boxes with untranslated regions in gray, and translated regions in white. SNPs denoted in light blue are located in the CTCF binding site, and in green are the tag SNPs (correlation coefficient: r^2^>0.85, minor allele frequency: MAF>0.10).

**Fig 2:**
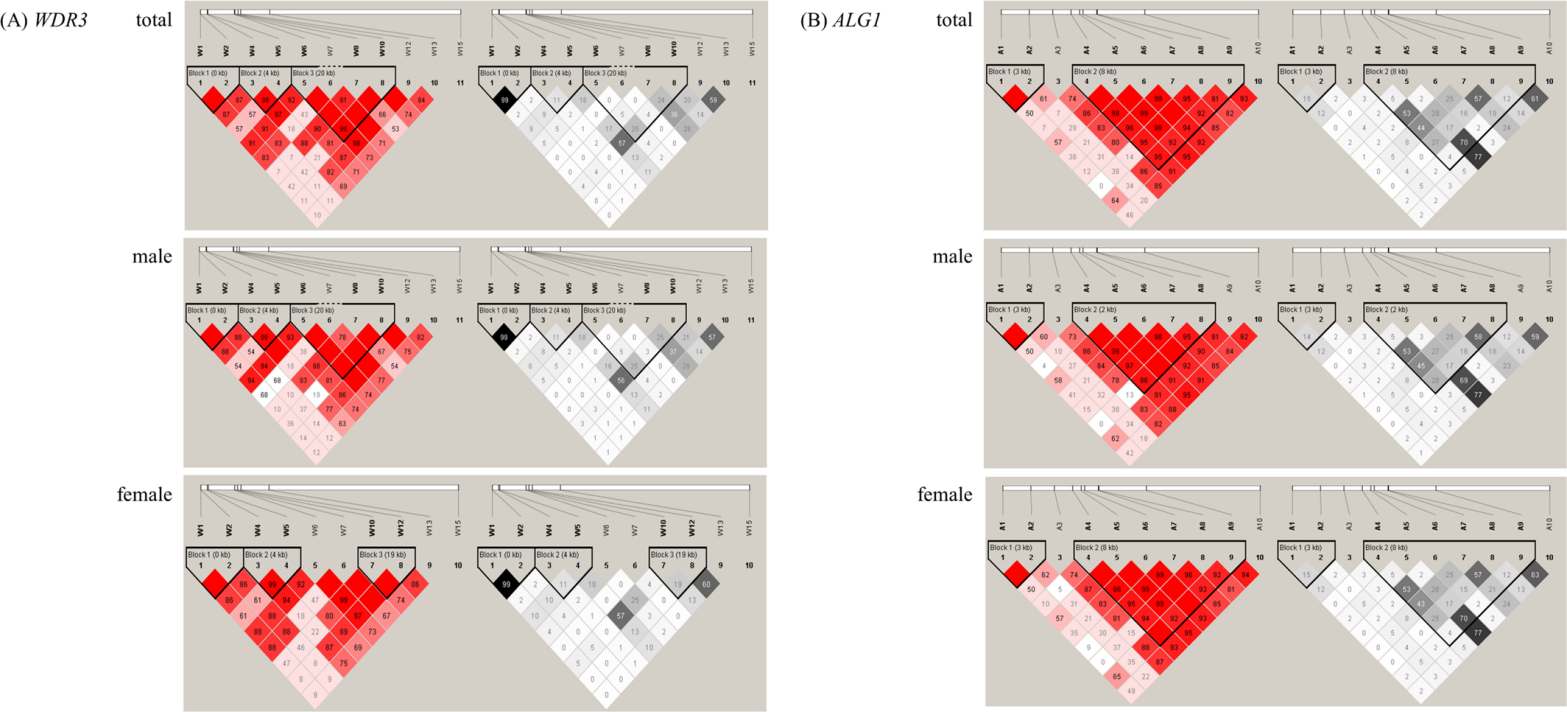
LD block structure of *WDR3* and *ALG1* genes. (A) *WDR3* gene consists of three, and (B) *ALG1* gene consists of two haplotype blocks in schizophrenia. In the left panel, the number in the box represents D’ (×100), blank means D’ = 1. In the right panel, the number in the box represents r^2^ (×100).

In this study, we selected 26 SNPs (16 SNPs from *WDR3* and 10 SNPs from *ALG1*). A schematic representation of the structures of the human *WDR3* and *ALG1* genes and location of the SNPs are shown in **Fig 1** and **Table 1**. The LD block structures are shown in **Fig 2**. Five of the *WDR3* SNPs were excluded from the subsequent analysis; two SNPs due to unclear clustering by the TaqMan Assay [W3 (rs1469919) and W9 (rs6696092)], one SNP due to monomorphism [W16 (rs6656360)], and two SNPs due to significant deviations from the HWE in the controls [W11 (rs2295629) and W14 (rs3753262)]. Therefore, we examined 11 SNPs of the human *WDR3* gene and 10 SNPs of the human *ALG1* gene as the genetic association study of schizophrenia.

**Table 1:** SNP information for *WDR3* and *ALG1* genes.

***WDR3***						
**SNP ID**	**rs number**	**Major/minor**	**Strand**	**Location**	**Function**	**MAF**
W1	rs1812607	C/T	+	5' upstream region	INS	T = 0.1616/353
W2	rs965361	A/T	+	5' upstream region	INS	T = 0.1625/355
W3	rs1469919	C/T	-	5' upstream region	INS	T = 0.2798/611
W4	rs319471	C/T	-	5' upstream region	INS	T = 0.1529/334
W5	rs379058	T/A	+	5' upstream region	INS	A = 0.3608/788
W6	rs3754127	C/T	+	5' upstream region	tag	T = 0.2807/613
W7	rs17037749	A/C	+	5' upstream region	INS	C = 0.0412/90
W8	rs1321663	G/C	+	intron1	tag	C = 0.0971/212
W9	rs6696092	A/G	+	intron3	tag	G = 0.4318/943
W10	rs1321666	T/C	+	intron13	tag	C = 0.4881/1066
W11	rs2295629	G/A	+	intron14	tag	A = 0.1946/425
W12	rs10802003	G/C	+	3' downstream region	tag	C = 0.0536/117
W13	rs10754369	C/T	+	3' downstream region	INS	T = 0.0847/185
W14	rs3753262	A/T	-	3' downstream region	INS	A = 0.3571/780
W15	rs3753261	C/T	-	3' downstream region	INS	T = 0.0627/137
W16	rs6656360	G/A	+	3' downstream region	INS	A = 0.0394/86
***ALG1***						
**SNP ID**	**rs number**	**Major/minor**	**Strand**	**Location**	**Function**	**MAF**
A1	rs8053916	C/G	+	5' upstream region	tag	G = 0.204/446
A2	rs9924614	C/T	+	5' upstream region	tag	T = 0.254/555
A3	rs9932909	C/T	+	5' upstream region	tag	T = 0.436/953
A4	rs3760030	C/T	-	5' upstream region	tag	T = 0.349/762
A5	rs3760029	C/T	-	5' upstream region	tag	T = 0.088/192
A6	rs3760027	A/G	-	5' upstream region	tag	G = 0.272/594
A7	rs8045294	G/C	+	intron1	tag	C = 0.467/1020
A8	rs8045473	C/G	+	intron1	tag	G = 0.3567/779
A9	rs7195893	C/T	+	exon6	tag	T = 0.0856/187
A10	rs9673733	C/G	+	3' downstream region	tag	C = 0.1049/229

INS: insulator, MAF: minor allele frequency.

The allelic frequency and genotypic distributions of all the experimentally genotyped SNPs are summarized in **Table 2**. Two *ALG1* SNPs and one *WDR3* SNP showed a tendency of association at the level of P <0.05 [A9 (rs7195893) and A10 (rs9673733) in the allelic tests, W8 (rs1321663) in the genotypic test]. The block-based haplotype analysis is shown in **Table 3**. For the haplotype analysis, the *WDR3* block [W4 (rs319471)–W5 (rs379058)] and the *ALG1* block [A1 (rs8053916)–A2 (rs9924614)] showed a related trend at the Global and Individual *P*-value, respectively. However, these allelic, genotypic, and haplotypic associations did not remain after correction for multiple testing.

**Table 2:** Genotyping and allele distribution of SNPs on *WDR3* and *ALG1* genes in schizophrenia and controls from the Japanese population.

***WDR3***														
**SNP ID**	**Affection**	**N**	**HWE *P***	**Allele count**		**MAF**	**Allelic *P***	**(FDR *P*)**	**OR (95% CI)**	**Genotypic count**			**Genotypic *P***	**(FDR *P*)**
**rs number**														
W1	CON	2,168	0.086	C	T	0.216	0.446	(0.701)	1.043 (0.938–1.161)	CC	CT	TT	0.076	(0.220)
				3,400	936					1,319	762	87		
rs1812607	SCZ	1,806	0.222	2,806	806	0.223				1,099	608	99		
W2	CON	2,168	0.066	A	T	0.215	0.430	(0.701)	1.045 (0.932–1.62)	AA	AT	TT	0.080	(0.220)
				3,402	934					1,320	762	86		
rs965361	SCZ	1,808	0.277	2,810	806	0.223				1,100	610	98		
W4	CON	2,170	0.914	C	T	0.113	0.074	(0.413)	0.877 (0.759–1.012)	CC	CT	TT	0.200	(0.367)
				3,851	489					1,709	433	28		
rs319471	SCZ	1,807	0.794	3,252	362	0.100				1,464	324	19		
W5	CON	2,168	0.122	T	A	0.495	0.543	(0.710)	0.972 (0.890–1.062)	TT	TA	AA	0.681	(0.955)
				2,189	2,147					534	1,121	513		
rs379058	SCZ	1,808	0.541	1,851	1,765	0.488				467	917	424		
W6	CON	2,169	0.428	C	T	0.182	0.581	(0.710)	1.033 (0.922–1.158)	CC	CT	TT	0.840	(0.955)
				3,548	790					1,445	658	66		
rs3754127	SCZ	1,807	0.354	2,938	676	0.187				1,188	562	57		
W7	CON	2,169	0.530	A	C	0.036	0.904	(0.904)	0.979 (0.772–1.240)	AA	AC	CC	0.947	(0.955)
				4,180	158					2,015	150	4		
rs17037749	SCZ	1,808	0.286	3,487	129	0.036				1,683	121	4		
W8	CON	2,169	0.088	G	C	0.176	0.161	(0.590)	1.086 (0.969–1.217)	GG	GC	CC	**0.042**	(0.220)
				3,575	763					1,485	605	79		
rs1321663	SCZ	1,807	0.248	2,934	680	0.188				1,183	568	56		
W10	CON	2,167	0.697	T	C	0.463	0.075	(0.413)	1.085 (0.993–1.185)	TT	TC	CC	0.182	(0.367)
				2,328	2,006					630	1,068	469		
rs1321666	SCZ	1,804	0.888	1,865	1,743	0.483				480	905	419		
W12	CON	2,169	0.208	G	C	0.151	0.350	(0.701)	1.061 (0.939–1.198)	GG	GC	CC	0.069	(0.220)
				3,684	654					1,572	540	57		
rs10802003	SCZ	1,808	0.112	3,043	573	0.158				1,271	501	36		
W13	CON	2,170	0.884	C	T	0.179	0.431	(0.701)	1.047 (0.934–1.174)	CC	CT	TT	0.717	(0.955)
				3,563	777					1,461	641	68		
rs10754369	SCZ	1,807	0.756	2,942	672	0.186				1,195	552	60		
W15	CON	2,169	0.827	C	T	0.110	0.858	(0.904)	1.013 (0.881–1.166)	CC	CT	TT	0.955	(0.955)
				3,859	479					1,715	429	25		
rs3753261	SCZ	1,808	0.635	3,212	404	0.112				1,424	364	20		
***ALG1***														
**SNP ID**	**Affection**	**N**	**HWE *P***	**Allele count**		**MAF**	**Allelic *P***	**(FDR *P*)**	**OR (95% CI)**	**Genotypic count**			**Genotypic *P***	**(FDR *P*)**
**rs number**														
A1	CON	2,169	0.211	C	G	0.311	0.149	(0.373)	0.931 (0.846–1.025)	CC	GC	GG	0.319	(0.638)
				2,987	1,351					1,041	905	223		
rs8053916	SCZ	1,807	0.652	2,543	1,071	0.296				899	745	163		
A2	CON	2,170	0.656	C	T	0.260	0.314	(0.449)	0.948 (0.857–1.049)	CC	CT	TT	0.587	(0.652)
				3,211	1,129					1,192	827	151		
rs9924614	SCZ	1,808	0.531	2,712	904	0.250				1,022	668	118		
A3	CON	2,161	0.769	C	T	0.179	0.554	(0.612)	0.965 (0.859–1.084)	CC	TC	TT	0.837	(0.837)
				3,550	772					1,460	630	71		
rs9932909	SCZ	1,799	0.870	2,974	624	0.173				1,230	514	55		
A4	CON	2,165	0.543	C	T	0.229	0.612	(0.612)	1.029 (0.927–1.142)	CC	TC	TT	0.463	(0.652)
				3,337	993					1,291	755	119		
rs3760030	SCZ	1,807	0.359	2,767	847	0.234				1,052	663	92		
A5	CON	2,166	0.686	C	T	0.158	0.079	(0.263)	0.894 (0.790–1.012)	CC	TC	TT	0.204	(0.638)
				3,647	685					1,532	583	51		
rs3760029	SCZ	1,805	0.924	3,091	519	0.144				1,322	447	36		
A6	CON	2,147	0.415	T	C	0.138	0.399	(0.499)	1.057 (0.931–1.200)	TT	CT	CC	0.546	(0.652)
				3,701	593					1,590	521	36		
rs3760027	SCZ	1,795	0.924	3,070	520	0.145				1,313	444	38		
A7	CON	2,166	0.822	G	C	0.393	0.298	(0.449)	0.952 (0.870–1.043)	GG	CG	CC	0.275	(0.638)
				2,630	1,702					801	1,028	337		
rs8045294	SCZ	1,803	0.163	2,231	1,375	0.381				676	879	248		
A8	CON	2,166	0.636	C	G	0.493	0.232	(0.449)	1.056 (0.966–1.153)	CC	GC	GG	0.476	(0.652)
				2,197	2,135					551	1,095	520		
rs8045473	SCZ	1,804	0.851	1,781	1,827	0.506				437	907	460		
A9	CON	2,160	0.856	C	T	0.137	**0.018**	(0.180)	0.851 (0.745–0.973)	CC	TC	TT	0.055	(0.335)
				3,728	592					1,607	514	39		
rs7195893	SCZ	1,801	0.736	3,173	429	0.119				1,399	375	27		
A10	CON	2,170	0.563	C	G	0.182	**0.037**	(0.185)	0.882 (0.785–0.992)	CC	CG	GG	0.067	(0.335)
				3,552	788					1,449	654	67		
rs9673733	SCZ	1,808	0.439	3,024	592	0.164				1,269	486	53		

N: number of subjects, HWE: Hardy-Weinberg equilibrium, MAF: minor allele frequency, FDR: the false discovery rate using the Benjamini-Hochberg procedure, OR: odds ratio, 95% CI: 95% confidence interval, CON: control, SCZ: schizophrenia.

**Table 3:** Block-based haplotype analysis of *WDR3* and *ALG1* genes.

***WDR3***											
**Marker**						**Frequency**		**OR (95% CI)**	***P*-values**		
						**SCZ**	**CON**		**Individual *P***	**Global *P***	**FDR *P***
W1			W2								
C			A			0.777	0.784	0.957 (0.860–1.065)	0.441		
T			T			0.223	0.215	1.045 (0.939–1.163)	0.415	0.506	0.506
W4			W5								
C			A			0.488	0.494	1.101 (0.947–1.279)	0.575		
C			T			0.412	0.393	**0.199 (0.176–0.224)**	0.086		
T			T			0.100	0.112	0.885 (0.766–1.022)	0.087	**0.043**	0.128
W6		W8		W10							
C		C		C		0.188	0.176	1.087 (0.970–1.219)	0.153		
C		G		C		0.108	0.105	1.031 (0.894–1.190)	0.681		
C		G		T		0.517	0.537	0.924 (0.846–1.010)	0.079		
T		G		C		0.186	0.182	1.028 (0.918–1.153)	0.631	0.284	0.426
***ALG1***											
**Marker**						**Frequency**		**OR (95% CI)**	***P*-values**		
						**SCZ**	**CON**		**Individual *P***	**Global *P***	**FDR *P***
A1			A2								
C			C			0.454	0.429	**1.108 (1.014–1.211)**	**0.024**		
C			T			0.250	0.260	0.948 (0.857–1.049)	0.301		
G			C			0.296	0.311	0.931 (0.846–1.025)	0.146	0.077	0.154
A4	A5	A6	A7	A8	A9						
C	C	C	G	C	C	0.120	0.122	0.969 (0.845–1.111)	0.661		
C	C	C	G	G	C	0.489	0.474	1.055 (0.962–1.156)	0.171		
C	T	C	C	C	T	0.113	0.127	**0.867 (0.755–0.995)**	0.052		
T	C	C	C	C	C	0.084	0.085	0.991 (0.844–1.163)	0.981		
T	C	T	C	C	C	0.141	0.134	1.056 (0.927–1.203)	0.384	0.504	0.504

OR: odds ratio, 95% CI: 95% confidence interval, CON: control, SCZ: schizophrenia, FDR: the false discovery rate using the Benjamini-Hochberg procedure

For the gender-stratification analysis, the allelic and genotypic distributions of each SNP in the schizophrenic patients and controls are shown in **Table 4**. Among the males, all the SNPs did not show deviations from the HWE. On the other hand, among the females, *WDR3* SNP W8 (rs1321663) was omitted from the analysis due to a significant deviation from the HWE in the female controls. In the female schizophrenia patients, *WDR3* SNP W12 (rs10802003), *ALG1* SNP A4 (rs3760030) and A7 (rs8045294) showed significant deviations from the HWE (*P* = 0.023, 0.026 and 0.024, respectively). We carefully interpreted the results of these 3 SNPs in the females.

**Table 4:** Stratification analysis of sex on *WDR3* and *ALG1* gene in schizophrenia and controls from Japanese population.

***WDR3***																							
**SNP ID**	**Affection**	**N**		**HWE *P***		**Allele count**				**MAF**		**Allelic *P* (FDR *P*)**		**OR (95% CI)**		**Genotypic count**						**Genotypic *P* (FDR *P*)**	
**rs number**		**Male**	**Female**	**Male**	**Female**	**Male**		**Female**		**Male**	**Female**	**Male**	**Female**	**Male**	**Female**	**Male**			**Female**			**Male**	**Female**
						C	T	C	T							CC	CT	TT	CC	CT	TT		
W1	CON	887	1,281	0.211	0.272	1,374	400	2,026	536	0.225	0.209	0.529	0.099	0.951	1.135	525	324	38	794	438	49	0.242	0.082
rs1812607	SCZ	992	814	0.401	0.375	1,554	430	1,252	376	0.217	0.231	(0.845)	(0.218)	(0.8146–1.109)	(0.978–1.318)	613	328	51	486	280	48	(0.897)	(0.180)
						A	T	A	T							AA	AT	TT	AA	AT	TT		
W2	CON	887	1,281	0.211	0.235	1,374	400	2,028	534	0.225	0.208	0.503	0.084	0.948	1.141	525	324	38	795	438	48	0.277	0.067
rs965361	SCZ	992	816	0.512	0.376	1,555	429	1,255	377	0.216	0.231	(0.845)	(0.218)	(0.8121–1.106)	(0.983–1.325)	613	329	50	487	281	48	(0.897)	(0.180)
						C	T	C	T							CC	CT	TT	CC	CT	TT		
W4	CON	889	1,281	0.110	0.278	1,588	190	2,263	299	0.107	0.117	0.753	**0.003**	1.038	**0.727**	714	160	15	995	273	13	0.897	**0.008**
rs319471	SCZ	991	816	0.197	0.191	1,763	219	1,489	143	0.110	0.088	(0.845)	**(0.033)**	(0.8451–1.275)	**(0.589–0.897)**	788	187	16	676	137	3	(0.897)	(0.088)
						T	A	T	A							TT	TA	AA	TT	TA	AA		
W5	CON	888	1,280	0.591	0.131	879	879	1,292	1,268	0.500	0.495	0.281	0.681	0.930	1.026	222	453	213	312	668	300	0.355	0.849
rs379058	SCZ	992	816	0.484	0.093	1,038	946	813	819	0.477	0.502	(0.845)	(0.742)	(0.8182–1.057)	(0.907–1.162)	277	484	231	190	433	193	(0.897)	(0.849)
						C	T	C	T							CC	CT	TT	CC	CT	TT		
W6	CON	888	1,281	0.653	0.638	1,451	325	2,097	465	0.183	0.181	0.737	0.713	1.032	1.032	590	271	27	855	387	39	0.886	0.776
rs3754127	SCZ	991	816	0.917	0.249	1,610	372	1,328	304	0.188	0.186	(0.845)	(0.742)	(0.8748–1.216)	(0.880–1.212)	653	304	34	535	258	23	(0.897)	(0.849)
						A	C	A	C							AA	AC	CC	AA	AC	CC		
W7	CON	889	1,280	1.000	0.419	1,716	62	2,464	96	0.035	0.038	0.717	0.742	0.923	1.065	828	60	1	1,187	90	3	0.854	0.818
rs17037749	SCZ	992	816	1.000	0.130	1,920	64	1,567	65	0.032	0.040	(0.845)	(0.742)	(0.6467–1.316)	(0.772–1.468)	929	62	1	754	59	3	(0.897)	(0.849)
						G	C	G	C							GG	GC	CC	GG	GC	CC		
W8	CON	889	1,280	0.911	**0.029**	1,449	329	2,126	434	0.185	0.170	0.933	0.089	1.010	1.150	591	267	31	894	338	48	0.747	0.033
rs1321663	SCZ	992	815	0.402	0.495	1,614	370	1,320	310	0.186	0.190	(0.933)	(0.218)	(0.8564–1.190)	(0.979–1.352)	652	310	30	531	258	26	(0.897)	(0.121)
						T	C	T	C							TT	TC	CC	TT	TC	CC		
W10	CON	888	1,279	0.590	0.311	947	829	1,381	1,177	0.467	0.460	0.452	0.087	1.051	1.117	248	451	189	382	617	280	0.622	0.127
rs1321666	SCZ	989	815	0.848	0.624	1,030	948	835	795	0.479	0.488	(0.845)	(0.218)	(0.9248–1.195)	(0.986–1.265)	270	490	229	210	415	190	(0.897)	(0.233)
						G	C	G	C							GG	GC	CC	GG	GC	CC		
W12	CON	889	1,280	0.799	0.121	1,503	275	2,181	379	0.155	0.148	0.561	0.595	1.055	1.051	636	231	22	936	309	35	0.806	**0.017**
rs10802003	SCZ	992	816	0.907	**0.023**	1,663	321	1,380	252	0.162	0.154	(0.845)	(0.742)	(0.8852–1.257)	(0.884–1.249)	696	271	25	575	230	11	(0.897)	(0.094)
						C	T	C	T							CC	CT	TT	CC	CT	TT		
W13	CON	889	1,281	0.114	0.292	1,456	322	2,107	455	0.181	0.178	0.768	0.459	1.028	1.064	589	278	22	872	363	46	0.300	0.257
rs10754369	SCZ	991	816	0.674	0.357	1,615	367	1,327	305	0.185	0.187	(0.845)	(0.742)	(0.8707–1.213)	(0.907–1.250)	660	295	36	535	257	24	(0.897)	(0.404)
						C	T	C	T							CC	CT	TT	CC	CT	TT		
W15	CON	889	1,280	0.328	0.238	1,571	207	2,288	272	0.116	0.106	0.681	0.610	0.956	1.056	697	177	15	1,018	252	10	0.436	0.583
rs3753261	SCZ	992	816	0.524	1.000	1,762	222	1,450	182	0.112	0.112	(0.845)	(0.742)	(0.7819–1.169)	(0.865–1.288)	780	202	10	644	162	10	(0.897)	(0.802)
***ALG1***																							
**SNP ID**	**Affection**	**N**		**HWE *P***		**Allele count**				**MAF**		**Allelic P (FDR *P*)**		**OR (95% CI)**		**Genotypic count**						**Genotypic P (FDR *P*)**	
**rs number**		**Male**	**Female**	**Male**	**Female**	**Male**		**Female**		**Male**	**Female**	**Male**	**Female**	**Male**	**Female**	**Male**			**Female**			**Male**	**Female**
						C	G	C	G							CC	GC	GG	CC	GC	GG		
A1	CON	888	1,281	0.177	0.606	1,238	538	1,749	813	0.303	0.317	0.391	0.393	0.939	0.941	440	358	90	601	547	133	0.669	0.558
rs8053916	SCZ	992	815	0.316	0.741	1,409	575	1,134	496	0.290	0.304	(0.495)	(0.650)	(0.816–1.080)	(0.823–1.076)	507	395	90	392	350	73	(0.768)	(0.697)
						C	T	C	T							CC	CT	TT	CC	CT	TT		
A2	CON	889	1,281	0.732	0.825	1,302	476	1,909	653	0.268	0.255	0.264	0.636	0.919	0.965	479	344	66	713	483	85	0.403	0.482
rs9924614	SCZ	992	816	0.556	0.110	1,485	499	1,227	405	0.252	0.248	(0.495)	(0.752)	(0.794–1.064)	(0.836–1.114)	552	381	59	470	287	59	(0.768)	(0.697)
						C	T	C	T							CC	TC	TT	CC	TC	TT		
A3	CON	882	1,279	0.907	0.573	1,456	308	2,094	464	0.175	0.181	1	0.455	0.999	0.939	600	256	26	860	374	45	0.687	0.415
rs9932909	SCZ	989	810	0.270	0.388	1,633	345	1,341	279	0.174	0.172	(1)	(0.650)	(0.843–1.183)	(0.797–1.106)	679	275	35	551	239	20	(0.768)	(0.697)
						C	T	C	T							CC	TC	TT	CC	TC	TT		
A4	CON	884	1,281	0.344	1.000	1,360	408	1,977	585	0.231	0.228	0.337	0.677	1.077	0.967	528	304	52	763	451	67	0.623	0.155
rs3760030	SCZ	991	816	0.492	**0.026**	1,498	484	1,269	363	0.244	0.222	(0.495)	(0.752)	(0.926–1.252)	(0.833–1.122)	570	358	63	482	305	29	(0.768)	(0.610)
						C	T	C	T							CC	TC	TT	CC	TC	TT		
A5	CON	889	1,277	0.535	1.000	1,493	285	2,154	400	0.160	0.157	0.158	0.289	0.876	0.909	624	245	20	908	338	31	0.315	0.567
rs3760029	SCZ	991	814	1.000	0.888	1,698	284	1,393	235	0.143	0.144	(0.495)	(0.650)	(0.733–1.047)	(0.763–1.082)	727	244	20	595	203	16	(0.768)	(0.697)
						T	C	T	C							TT	CT	CC	TT	CT	CC		
A6	CON	880	1,267	0.888	0.475	1,514	246	2,187	347	0.140	0.137	0.307	1.000	1.101	0.997	650	214	16	940	307	20	0.391	0.918
rs3760027	SCZ	982	813	0.266	0.296	1,666	298	1,404	222	0.152	0.137	(0.495)	(1)	(0.917–1.321)	(0.831–1.195)	711	244	27	602	200	11	(0.768)	(0.918)
						G	C	G	C							GG	CG	CC	GG	CG	CC		
A7	CON	888	1,278	0.727	0.517	1,066	710	1,564	992	0.400	0.388	0.593	0.240	0.963	0.925	317	432	139	484	596	198	0.796	**0.043**
rs8045294	SCZ	989	814	0.894	**0.024**	1,205	773	1,026	602	0.391	0.370	(0.659)	(0.650)	(0.845–1.098)	(0.814–1.052)	368	469	152	308	410	96	(0.796)	(0.430)
						C	G	C	G							CC	GC	GG	CC	GC	GG		
A8	CON	888	1,278	0.503	0.240	908	868	1,267	1,289	0.489	0.504	0.396	0.358	1.059	0.943	237	434	217	303	661	314	0.691	0.627
rs8045473	SCZ	991	813	0.446	0.233	985	997	830	796	0.503	0.490	(0.495)	(0.650)	(0.932–1.204)	(0.832–1.068)	251	483	257	203	424	186	(0.768)	(0.697)
						C	T	C	T							CC	TC	TT	CC	TC	TT		
A9	CON	885	1,275	0.673	0.905	1,524	246	2,204	346	0.139	0.136	0.056	0.157	0.829	0.872	654	216	15	953	298	24	0.121	0.364
rs7195893	SCZ	987	814	0.648	0.870	1,741	233	1,432	196	0.118	0.120	(0.495)	(0.650)	(0.684–1.005)	(0.723–1.052)	769	203	15	630	172	12	(0.768)	(0.697)
						C	G	C	G							CC	CG	GG	CC	CG	GG		
A10	CON	889	1,281	0.911	0.397	1,453	325	2,099	463	0.183	0.181	0.196	0.094	0.892	0.867	594	265	30	855	389	37	0.363	0.183
rs9673733	SCZ	992	816	0.423	0.795	1,654	330	1,370	262	0.166	0.161	(0.495)	(0.650)	(0.754–1.056)	(0.734–1.024)	693	268	31	576	218	22	(0.768)	(0.610)

N: number of subjects, HWE: Hardy-Weinberg equilibrium, MAF: minor allele frequency, FDR: the false discovery rate using the Benjamini-Hochberg procedure, OR: odds ratio, 95% CI: 95% confidence interval, CON: control, SCZ: schizophrenia

As shown in **Table 4**, among the females, *WDR3* SNP W4 (rs319471) exhibited a significant allelic association in the female schizophrenic patients compared to the female controls [the C allele is overrepresented in the patients; *P* = 0.003; odds ratio (OR), 95% confidence interval (95% CI) = 1.38, 1.12–1.70]. This association remained even after correction for multiple testing (*P* = 0.033). *WDR3* SNP W4 (rs319471), W12 (rs10802003) and *ALG1* SNP A7 (rs8045294) also displayed a tendency to genotypic association in the female subjects with schizophrenia compared to the female controls, however, it was not significant after multiple testing (**Table 4**).

As displayed in **Table 5**, in the haplotype analysis, the block range from W4 (rs319471) to W5 (rs379058) showed a significant association in the female subjects with schizophrenia compared to the female controls (global haplotypic *P* = 0.016), even after correcting for the multiple testing; T [W4 (rs319471)]–T [W5 (rs379058)] is overrepresented in the controls (*P* = 0.003; OR, 95% CI = 0.731, 0.592–0.901). We did not observe such an association in the male schizophrenics compared to the male controls.

**Table 5:** Sex stratified block-based haplotype analysis of *WDR3* and *ALG1* genes.

**(A) *WDR3***											
Male											
**Marker**						**Frequency**		**OR (95% CI)**	***P*-values**		
						**SCZ**	**CON**		**Individual *P***	**Global *P***	**FDR *P***
W1			W2								
C			A			0.783	0.774	1.053 (0.903–1.229)	0.507		
T			T			0.216	0.225	0.949 (0.813–1.108)	0.509	0.801	0.870
W4			W5								
C			A			0.477	0.493	0.933 (0.821–1.061)	0.297		
C			T			0.413	0.400	1.051 (0.923–1.198)	0.399		
T			T			0.111	0.105	1.055 (0.858–1.298)	0.648	0.253	0.759
W6		W8		W10							
C		C		C		0.187	0.186	1.009 (0.855–1.189)	0.920		
C		G		C		0.105	0.099	1.069 (0.865–1.322)	0.537		
C		G		T		0.521	0.533	0.953 (0.838–1.084)	0.462		
T		G		C		0.187	0.183	1.031 (0.874–1.216)	0.717	0.870	0.870
Female											
**Marker**						**Frequency**		**OR (95% CI)**	***P*-values**		
						**SCZ**	**CON**		**Individual *P***	**Global *P***	**FDR *P***
W1			W2								
C			A			0.769	0.791	0.878 (0.756–1.019)	0.096		
T			T			0.231	0.208	1.139 (0.981–1.323)	0.085	0.087	0.131
W4			W5								
C			A			0.502	0.495	1.028 (0.908–1.164)	0.660		
C			T			0.411	0.389	1.094 (0.964–1.242)	0.152		
T			T			0.088	0.116	**0.731 (0.592–0.901)**	**0.003**	**0.016**	**0.048**
W10			W12								
C			C			0.155	0.148	1.054 (0.886–1.253)	0.554		
C			G			0.333	0.312	1.101 (0.964–1.257)	0.157		
T			G			0.512	0.540	0.895 (0.790–1.014)	0.081	0.213	0.213
**(B) *ALG1***											
Male											
**Marker**						**Frequency**		**OR (95% CI)**	***P*-values**		
						**SCZ**	**CON**		**Individual *P***	**Global *P***	**FDR *P***
A1			A2								
C			C			0.459	0.430	1.125 (0.989–1.280)	0.074		
C			T			0.252	0.268	0.92 (0.795–1.065)	0.265		
G			C			0.290	0.303	0.939 (0.816–1.080)	0.379	0.198	0.395
A4	A5	A6	A7	A8							
C	C	C	G	C		0.114	0.121	0.924 (0.756–1.129)	0.445		
C	C	C	G	G		0.489	0.477	1.044 (0.917–1.189)	0.520		
C	T	C	C	C		0.145	0.159	0.89 (0.743–1.065)	0.203		
T	C	C	C	C		0.087	0.088	0.992 (0.790–1.247)	0.946		
T	C	T	C	C		0.150	0.136	1.114 (0.926–1.340)	0.255	0.523	0.523
Female											
**Marker**						**Frequency**		**OR (95% CI)**	***P*-values**		
						**SCZ**	**CON**		**Individual *P***	**Global *P***	**FDR *P***
A1			A2								
C			C			0.448	0.428	1.085 (0.957–1.230)	0.202		
C			T			0.248	0.255	0.963 (0.835–1.112)	0.610		
G			C			0.304	0.317	0.941 (0.823–1.076)	0.375	0.437	0.720
A4	A5	A6	A7	A8	A9						
C	C	C	G	C	C	0.126	0.124	1.008 (0.834–1.219)	0.939		
C	C	C	G	G	C	0.496	0.475	1.074 (0.944–1.222)	0.288		
C	T	C	C	C	T	0.114	0.126	0.882 (0.726–1.072)	0.207		
T	C	C	C	C	C	0.083	0.084	0.978 (0.779–1.228)	0.853		
T	C	T	C	C	C	0.131	0.133	0.975 (0.809–1.174)	0.791	0.720	0.720

OR: odds ratio, 95% CI: 95% confidence interval, CON: control, SCZ: schizophrenia, FDR: the false discovery rate using the Benjamini-Hochberg procedure

Based on the age-at-onset stratification analysis, three of the *WDR3* SNPs [W4 (rs319471), W8 (rs1321663) and W12 (rs10802003)] and four of the *ALG1* SNPs [A1 (rs8053916), A5 (rs3760029), A6 (rs3760027) and A9 (rs7195893)] displayed a tendency to correlation with the different onset age groups of schizophrenia, although it was not significant after multiple testing (**S1 Table**).

By classifying the onset age groups of the male and female (**S2 Table**), five of the *WDR3* SNPs [W1 (rs1812607), W2 (rs965361), W4 (rs319471), W12 (rs10802003) and W13 (rs10754369)] exhibited a tendency to correlation in several of the onset age groups, although did not remain significant after multiple testing. In addition, these SNPs have a commonality that the onset-aged between the 26 and 35 year groups in the male and female schizophrenics. In the case sample, three of the *WDR3* SNPs [W4 (rs319471), W7 (rs17037749) and W13 (rs10754369)] showed a slight deviation from the HWE in the specific onset age groups of the males and females (16–25 years old in the males: *P* = 0.010, over 36 years old in the females: *P* = 0.040, over 36 years old in the females: *P* = 0.022, respectively). Three of the *ALG1* SNPs [A1 (rs8053916), A4 (rs3760030) and A7 (rs8045294)], although not significant after multiple testing, showed a tendency to correlation. Moreover, in the case sample, A4 (rs3760030) showed a slight deviation from the HWE in specific groups (26–35 years old in the females; *P* = 0.006). We further cautiously interpreted the results of the SNPs deviating from the HWE in the males and females.

### Gene-gene interaction analysis

Based on the MDR analysis, five of the *WDR3* SNPs were excluded for the same reasons as for the case-control Fisher’s exact test: W3 (rs1469919), W9 (rs6696092), W11 (rs2295629), W14 (rs3753262) and W16 (rs6656360). The LD block which consisted of W1 (rs1812607) -W2 (rs965361) showed a high LD (r^2^>0.95). Therefore, we searched the tag SNP for avoid any false evaluation. Using the HaploView program to examine the tag SNP, W1 (rs1812607) was detected. Therefore, SNP W2 (rs965361) was omitted. For the sex stratified analysis, the same six SNPs were excluded due to same reasons in the males. In the females, *WDR3* SNP W8 (rs1321663) was additionally excluded for the same reasons as for the case-control Fisher’s exact test. Therefore, 10 *ALG1* SNPs and 10 *WDR3* SNPs were analyzed in all the samples of the case-controls and male case-control samples. For the females, 10 *ALG1* SNPs and 9 *WDR3* SNPs were analyzed.

The testing accuracy (TA) represents the average value of the sensitivity and specificity. A TA of 0.55 and greater means that the MDR model is typically statistically significant. The best *P*-value was the combination of *ALG1* SNP A9 (rs7195893) and *WDR3* SNP W10 (rs1321666) in the female schizophrenia (*P* = 0.047), but the TA of this model was less than 0.55 (TA = 0.543). The chi-square *P*-value supported this result (*P* = 0.208). Therefore, it was not enough to indicate the interaction of these genes (**Table 6**).

**Table 6:** The MDR analysis for the best determined model.

Total							
**model**	**TA**	**CVC**	**Permutation *P***		**χ2**	***P***	**FDR *P***
			**TA**	**CVC**			
WD-08 (rs1321663)	0.499	4/10	0.868	0.947	0.001	0.978	0.978
AL-10 (rs9673733), WD-10 (rs1321666)	0.478	2/10	0.999–1.000	0.999–1.000	0.759	0.384	0.978
AL-01 (rs8053916), AL-08 (rs8045473), WD-08 (rs1321663)	0.506	4/10	0.665	0.947	0.062	0.803	0.978
AL-02 (rs9924614), AL-07 (rs8045294), AL-10 (rs9673733), WD-10 (rs1321666)	0.501	2/10	0.834	0.999–1.000	0.001	0.973	0.978
Male							
**model**	**TA**	**CVC**	**Permutation *P***		**χ2**	***P***	**FDR *P***
			**TA**	**CVC**			
AL-09 (rs7195893)	0.498	7/10	0.886	0.621	0.003	0.960	0.960
WD-04 (rs319471), WD-12 (rs10802003)	0.518	9/10	0.453	0.349	0.262	0.609	0.960
AL-01 (rs8053916), AL-09 (rs7195893), WD-13 (rs10754369)	0.490	2/10	0.964–0.965	0.999–1.000	0.077	0.781	0.960
AL-01 (rs8053916), AL-03 (rs9932909), AL-08 (rs8045473), WD-08 (rs1321663)	0.488	3/10	0.972–0.973	0.990	0.106	0.745	0.960
Female							
**model**	**TA**	**CVC**	**Permutation *P***		**χ2**	***P***	**FDR *P***
			**TA**	**CVC**			
WD-04 (rs319471)	0.511	8/10	0.623	0.493	0.123	0.726	0.726
AL-09 (rs7195893), WD-10 (rs1321666)	0.543	10/10	0.047	0.212	1.584	0.208	0.726
AL-10 (rs9673733), WD-05 (rs379058), WD-10 (rs1321666)	0.516	2/10	0.516	0.999–1.000	0.215	0.643	0.726
AL-02 (rs9924614), AL-07 (rs8045294), WD-05 (rs379058), WD-10 (rs1321666)	0.516	4/10	0.519–0.520	0.962	0.207	0.649	0.726

TA: testing accuracy, CVC: cross-validation consistency, FDR: the false discovery rate using the Benjamini-Hochberg procedure.

### Power estimation

The power analysis showed a 99.09% power in the genotypic test and a 99.64% power in the allelic test for the case-control statistics in our sample. Based on the stratified analysis according to sex, the powers of the female and male groups were 85.48% and 82.51% in the genotypic test and 91.3% and 89.02% in the allelic test, respectively. The other stratified groups consisting of the classified age at onset are shown in **Table 7**.

**Table 7:** Power estimation of case-control sample and classified groups.

Total						
	N		Genotypic		Allelic	
	SCZ	CON	Power (%)	N (80%)	Power (%)	N (80%)
Under 17	264	2170	53.92	468	64.42	381
Over 18	1426		98.1	719	99.18	589
Under 15	107		26.15	434	33.78	353
16–25	918		93.63	610	96.72	498
26–35	461		75.49	511	83.79	417
Over 36	204		44.41	455	54.67	371
Male						
	N		Genotypic		Allelic	
	SCZ	CON	Power (%)	N (80%)	Power (%)	N (80%)
Under 15	50	889	14.18	437	18.26	356
16–25	519		67.46	685	76.89	560
26–35	252		45.65	544	55.94	444
Over 36	94		22.22	460	28.87	375
Female						
	N		Genotypic		Allelic	
	SCZ	CON	Power (%)	N (80%)	Power (%)	N (80%)
Under 15	57	1281	15.73	431	20.36	351
16–25	399		64.79	558	74.62	455
26–35	209		42.68	488	52.81	397
Over 36	110		25.88	451	33.46	367

N: number of subjects, N (80%): Number that reaches 80% detection power, CON: control, SCZ: schizophrenia

## Discussion

This is the first genetic study of the *WDR3* and *ALG1* genes in schizophrenia to the best of our knowledge. We detected related signals between the *WDR3* genes and female schizophrenic patients. In the allelic tests, W4 (rs319471) indicated a significant association with schizophrenia among the female schizophrenia patients. In our block-based haplotype analysis, the block range from W4 (rs319471) to W5 (rs379058) exhibited a significant association in the female schizophrenics. In these analyses, no association was detected in the male or the group of all subjects. Indeed, gender differences related to schizophrenia have been widely known [32]. For example, the clinical observations showed that male patients were inclined to have earlier onset and a more severe course than female patients. In addition, male schizophrenics have more negative symptoms and cognitive deficits, while female schizophrenics show more affective symptoms [33]. For the molecular biological approaches, several genes that have sex-specific genetic associations with schizophrenia were reported such as Disrupted in Schizophrenia 1 (*DISC1*), reelin (*RELN*), D-amino acid oxidase (*DAO*) and synapse-associated protein 97/discs, large homolog 1 of Drosophila (*DLG1*) in previous studies [34–37]. Therefore, the female specific association revealed in the *WDR3* gene might be involved in the molecular basis of the schizophrenic pathology.

*WDR3* SNP W4 (rs319471), which is located in the CTCF binding site of the 5’ upstream of the *WDR3* gene, showed a significant association with female schizophrenics. This study focused on the CTCF binding site to select the SNPs as the gene expression control by the insulator function. This function is well known to enhancer-blocking activity and as a barrier to chromosomal position effects [38]. Consequently, the polymorphism of this site might be linked to the insulator function/dysfunction of the *WDR3* and flanking cluster genes. We searched the sequence that contains 50 base pairs up- and down-stream of W4 (rs319471) at CTCFBSDB 2.0; a database for CTCF binding sites and genome organization (http://insulatordb.uthsc.edu/) [25, 39]. As a result, only when the SNP consists of the C allele does the sequence (ATCACTGCC) closely conform to the CTCF consensus. It might influence the expression level. We searched the expression quantitative trait loci (eQTLs) using the Brain eQTL Almanac (http://www.braineac.org/) to investigate whether W4 (rs319471) affects the expression of WDR3 in the females. The change in the expression level was reported in a multitude of genes, however, there is no significant report on the expression level of *WDR3* in the database. Although we have to carefully consider that the database does not categorize the data by sex. To estimate the difference in the expression level in the female schizophrenics, the data by sex are needed. If it formed a CTCF consensus, the possibility is considered that the minor allele frequency is lower than in the healthy control at the female W4 (rs319471, minor allele: T), thus it is possible that the CTCF-binding activity is higher in schizophrenia. Moreover, based on a block-based haplotype analysis, the block consisting of W4 (rs319471) showed a significant correlation in female schizophrenia. It may support the fact that W4 (rs319471) is located in the disease susceptibility region. Actually, the influence of the CTCF-binding activity in the brain was reported. The CTCF-deficient neuron showed defects in the dendritic arborization and spine density during brain development [40]. Additionally, a decline in the cohesin function in the brain leads to a defective synapse development and anxiety-related behavior [41]. This means that the CTCF-binding activity has relevance to functional neural development and neuronal diversity. Accordingly, W4 (rs319471) has the possibilities involved in the pathophysiology of schizophrenia via the chromatin conformational changes.

The *ALG1* SNPs showed only a statistically-weak correlation, however, two SNPs [A4 (rs3760030) and A7 (rs8045294)] that showed a tendency of association with female schizophrenia were reported as the eQTLs [42, 43]. Furthermore, the chromosome 16p13 region, *ALG1* located, was reported to have copy number variations associated with schizophrenia [44]. The *ALG1* gene did not show a strong correlation with schizophrenia in this study, however, the SNPs that showed a trend associated with a specific onset-age groups were observed. This may suggest that the SNPs or there genomic region affects the onset age of schizophrenia.

As for the age-at-onset analysis, there was no statistically significant association. This may be explained by the low statistical power in our stratified age-at-onset groups (<80%). Therefore, a larger sample size group needs to be studied to use the age-at-onset analysis.

In conclusion, our present associations study demonstrated that the *WDR3* gene is selectively related to female schizophrenia. These results indicated that the *WDR3* gene may be a susceptibility factor in female subjects with schizophrenia, and that regulation of the *WDR3* signaling pathway ensures further research from the aspect of the pathophysiology of schizophrenia.

Further study is required to elucidate the gender-dependent correlation between the *WDR3* gene and schizophrenia using different ethnic populations and larger sample sizes.

## Supporting information

S1 Table Stratification analysis of onset-age groups on *WDR3* and *ALG1* genes in schizophrenia and controls from Japanese population. N: number of subjects, HWE: Hardy-Weinberg equilibrium, MAF: minor allele frequency, FDR: the false discovery rate using the Benjamini-Hochberg procedure, OR: odds ratio, 95% CI: 95% confidence interval, CON: control, SCZ: schizophrenia.
(PDF)

S2 Table Stratification analysis of onset-age groups by sex on *WDR3* and *ALG1* genes in schizophrenia and controls from Japanese population. N: number of subjects, HWE: Hardy-Weinberg equilibrium, MAF: minor allele frequency, FDR: the false discovery rate using the Benjamini-Hochberg procedure, OR: odds ratio, 95% CI: 95% confidence interval, CON: control, SCZ: schizophrenia.
(PDF)

## References

[1] MacDonald AW, Schulz SC. What we know: findings that every theory of schizophrenia should explain. Schizophr Bull. 2009 5;35(3):493–508. doi: 10.1093/schbul/sbp017 19329559 10.1093/schbul/sbp017 PMC2669587

[2] Yui K, Goto K, Ikemoto S, Ishiguro T, Angrist B, Duncan GE, et al Neurobiological basis of relapse prediction in stimulant-induced psychosis and schizophrenia: the role of sensitization. Mol Psychiatry. 1999 11;4(6):512–23. 10578232 10.1038/sj.mp.4000575

[3] Reich DL, Silvay G. Ketamine: an update on the first twenty-five years of clinical experience. Can J Anaesth. 1989 3;36(2):186–97. doi: 10.1007/BF03011442 2650898 10.1007/BF03011442

[4] White PF, Way WL, Trevor AJ. Ketamine—its pharmacology and therapeutic uses. Anesthesiology. 1982 2;56(2):119–36. 6892475 10.1097/00000542-198202000-00007

[5] Fujiwara Y, Kazahaya Y, Nakashima M, Sato M, Otsuki S. Behavioral sensitization to methamphetamine in the rat: an ontogenic study. Psychopharmacology (Berl). 1987;91(3):316–9. 3104953 10.1007/BF00518183

[6] Scalzo FM, Burge LJ. The role of NMDA and sigma systems in the behavioral effects of phencyclidine in preweanling rats. Neurotoxicology. 1994 Spring;15(1):191–200. 8090359

[7] Scalzo FM, Holson RR. The ontogeny of behavioral sensitization to phencyclidine. Neurotoxicol Teratol. 1992 Jan-Feb;14(1):7–14. 1593982 10.1016/0892-0362(92)90023-4

[8] Nishikawa T, Umino A, Kashiwa A, Ooshima A, Nomura N, Takahashi K. Stimulant-induced behavioral sensitization and cerebral neurotransmission In: Toru M (ed). Neurotransmitters in Neuronal Plasticity and Psychiatric Disorders. Excerpta Medica: Tokyo, 1993, pp 53–62.

[9] Sato D, Umino A, Kaneda K, Takigawa M, Nishikawa T. Developmental changes in distribution patterns of phencyclidine-induced c-Fos in rat forebrain. Neurosci Lett. 1997 12 12;239(1):21–4. 9547162 10.1016/s0304-3940(97)00879-3

[10] Lewis CM, Levinson DF, Wise LH, DeLisi LE, Straub RE, Hovatta I, et al Genome scan meta-analysis of schizophrenia and bipolar disorder, part II: Schizophrenia. Am J Hum Genet. 2003 7;73(1):34–48. Epub 2003 Jun 11. doi: 10.1086/376549 12802786 10.1086/376549 PMC1180588

[11] Ng MY, Levinson DF, Faraone SV, Suarez BK, DeLisi LE, Arinami T, et al Meta-analysis of 32 genome-wide linkage studies of schizophrenia. Mol Psychiatry. 2009 8;14(8):774–85. doi: 10.1038/mp.2008.135 19349958 10.1038/mp.2008.135 PMC2715392

[12] Claudio JO, Liew CC, Ma J, Heng HH, Stewart AK, Hawley RG. Cloning and expression analysis of a novel WD repeat gene, WDR3, mapping to 1p12-p13. Genomics. 1999 7 1;59(1):85–9. doi: 10.1006/geno.1999.5858 10395803 10.1006/geno.1999.5858

[13] Zhang Cheng, Lin Jinzhong, Liu Weixiao, Chen Xining, Chen Rongchang, Ye Keqiong. Structure of Utp21 Tandem WD Domain Provides Insight into the Organization of the UTPB Complex Involved in Ribosome Synthesis. PLoS One. 2014 1 21;9(1):e86540 doi: 10.1371/journal.pone.0086540 24466140 10.1371/journal.pone.0086540 PMC3897721

[14] McMahon M, Ayllón V, Panov KI, O'Connor R. Ribosomal 18 S RNA processing by the IGF-I-responsive WDR3 protein is integrated with p53 function in cancer cell proliferation. J Biol Chem. 2010 6 11;285(24):18309–18. doi: 10.1074/jbc.M110.108555 20392698 10.1074/jbc.M110.108555 PMC2881756

[15] Grubenmann CE, Frank CG, Hülsmeier AJ, Schollen E, Matthijs G, Mayatepek E, et al Deficiency of the first mannosylation step in the N-glycosylation pathway causes congenital disorder of glycosylation type Ik. Hum Mol Genet. 2004 3 1;13(5):535–42. doi: 10.1093/hmg/ddh050 14709599 10.1093/hmg/ddh050

[16] Bauer D, Haroutunian V, Meador-Woodruff JH, McCullumsmith RE. Abnormal glycosylation of EAAT1 and EAAT2 in prefrontal cortex of elderly patients with schizophrenia. Schizophr Res. 2010 3;117(1):92–8. doi: 10.1016/j.schres.2009.07.025 19716271 10.1016/j.schres.2009.07.025 PMC2822023

[17] Yamaguchi-Kabata Y, Nakazono K, Takahashi A, Saito S, Hosono N, Kubo M, et al Japanese population structure, based on SNP genotypes from 7003 individuals compared to other ethnic groups: effects on population-based association studies. Am J Hum Genet. 2008 10;83(4):445–56. doi: 10.1016/j.ajhg.2008.08.019 18817904 10.1016/j.ajhg.2008.08.019 PMC2561928

[18] Hattori E, Toyota T, Ishitsuka Y, Iwayama Y, Yamada K, Ujike H, et al Preliminary genome-wide association study of bipolar disorder in the Japanese population. Am J Med Genet B Neuropsychiatr Genet. 2009 12 5;150B(8):1110–7. doi: 10.1002/ajmg.b.30941 19259986 10.1002/ajmg.b.30941

[19] Pritchard JK, Stephens M, Donnelly P. Inference of population structure using multilocus genotype data. Genetics. 2000 6;155(2):945–59. 10835412 10.1093/genetics/155.2.945 PMC1461096

[20] Yamada K, Nakamura K, Minabe Y, Iwayama-Shigeno Y, Takao H, Toyota T, et al Association analysis of FEZ1 variants with schizophrenia in Japanese cohorts. Biol Psychiatry. 2004 11 1;56(9):683–90. doi: 10.1016/j.biopsych.2004.08.015 15522253 10.1016/j.biopsych.2004.08.015

[21] Yamada K, Hattori E, Iwayama Y, Ohnishi T, Ohba H, Toyota T, et al Distinguishable haplotype blocks in the HTR3A and HTR3B region in the Japanese reveal evidence of association of HTR3B with female major depression. Biol Psychiatry. 2006 7 15;60(2):192–201. doi: 10.1016/j.biopsych.2005.11.008 16487942 10.1016/j.biopsych.2005.11.008

[22] Devlin B, Roeder K. Genomic control for association studies. Biometrics. 1999 12;55(4):997–1004. 11315092 10.1111/j.0006-341x.1999.00997.x

[23] Balan S, Yamada K, Hattori E, Iwayama Y, Toyota T, Ohnishi T, et al Population-specific haplotype association of the postsynaptic density gene DLG4 with schizophrenia, in family-based association studies. PLoS One. 2013 7 25;8(7):e70302 doi: 10.1371/journal.pone.0070302 23936182 10.1371/journal.pone.0070302 PMC3723755

[24] Carlson CS, Eberle MA, Rieder MJ, Yi Q, Kruglyak L, Nickerson DA. Selecting a maximally informative set of single-nucleotide polymorphisms for association analyses using linkage disequilibrium. Am J Hum Genet. 2004 1;74(1):106–20. doi: 10.1086/381000 14681826 10.1086/381000 PMC1181897

[25] Ziebarth JD, Bhattacharya A, Cui Y. CTCFBSDB 2.0: a database for CTCF-binding sites and genome organization. Nucleic Acids Res. 2013 1;41(Database issue):D188–94. doi: 10.1093/nar/gks1165 23193294 10.1093/nar/gks1165 PMC3531215

[26] Purcell S, Cherny SS, Sham PC. Genetic power calculator: design of linkage and association genetic mapping studies of complex traits. Bioinformatics. 2003 1;19(1):149–50. 12499305 10.1093/bioinformatics/19.1.149

[27] Barrett JC, Fry B, Maller J, Daly MJ. Haploview: analysis and visualization of LD and haplotype maps. Bioinformatics. 2005 1 15;21(2):263–5. doi: 10.1093/bioinformatics/bth457 15297300 10.1093/bioinformatics/bth457

[28] Gabriel SB, Schaffner SF, Nguyen H, Moore JM, Roy J, Blumenstiel B, et al The structure of haplotype blocks in the human genome. Science. 2002 6 21;296(5576):2225–9. doi: 10.1126/science.1069424 12029063 10.1126/science.1069424

[29] Clemmensen L, Vernal DL, Steinhausen HC. A systematic review of the long-term outcome of early onset schizophrenia. BMC Psychiatry. 2012 9 19;12:150 doi: 10.1186/1471-244X-12-150 22992395 10.1186/1471-244X-12-150 PMC3521197

[30] Moore JH1, Andrews PC. Epistasis analysis using multifactor dimensionality reduction. Methods Mol Biol. 2015;1253:301–14. doi: 10.1007/978-1-4939-2155-3_16 25403539 10.1007/978-1-4939-2155-3_16

[31] Hahn LW, Ritchie MD, Moore JH. Multifactor dimensionality reduction software for detecting gene-gene and gene-environment interactions. Bioinformatics. 2003 2 12;19(3):376–82. 12584123 10.1093/bioinformatics/btf869

[32] Leung A, Chue P. Sex differences in schizophrenia, a review of the literature. Acta Psychiatr Scand Suppl. 2000;401:3–38. 10887978 10.1111/j.0065-1591.2000.0ap25.x

[33] Mendrek A, Mancini-Marïe A. Sex/gender differences in the brain and cognition in schizophrenia. Neurosci Biobehav Rev. 2016 8;67:57–78. doi: 10.1016/j.neubiorev.2015.10.013 26743859 10.1016/j.neubiorev.2015.10.013

[34] Hennah W, Varilo T, Kestilä M, Paunio T, Arajärvi R, Haukka J, et al Haplotype transmission analysis provides evidence of association for DISC1 to schizophrenia and suggests sex-dependent effects. Hum Mol Genet. 2003 12 1;12(23):3151–9. doi: 10.1093/hmg/ddg341 14532331 10.1093/hmg/ddg341

[35] Shifman S, Johannesson M, Bronstein M, Chen SX, Collier DA, Craddock NJ, et al Genome-wide association identifies a common variant in the reelin gene that increases the risk of schizophrenia only in women. PLoS Genet. 2008 2;4(2):e28 doi: 10.1371/journal.pgen.0040028 18282107 10.1371/journal.pgen.0040028 PMC2242812

[36] Kim B, Kim H, Joo YH, Lim J, Kim CY, Song K. Sex-different association of DAO with schizophrenia in Koreans. Psychiatry Res. 2010 9 30;179(2):121–5. doi: 10.1016/j.psychres.2008.08.009 20483168 10.1016/j.psychres.2008.08.009

[37] Uezato A, Kimura-Sato J, Yamamoto N, Iijima Y, Kunugi H, Nishikawa T. Further evidence for a male-selective genetic association of synapse-associated protein 97 (SAP97) gene with schizophrenia. Behav Brain Funct. 2012 1 6;8:2 doi: 10.1186/1744-9081-8-2 22225629 10.1186/1744-9081-8-2 PMC3275478

[38] Wendt KS, Yoshida K, Itoh T, Bando M, Koch B, Schirghuber E, et al Cohesin mediates transcriptional insulation by CCCTC-binding factor. Nature. 2008 2 14;451(7180):796–801. doi: 10.1038/nature06634 18235444 10.1038/nature06634

[39] Bao L, Zhou M, Cui Y. CTCFBSDB: a CTCF-binding site database for characterization of vertebrate genomic insulators. Nucleic Acids Res. 2008 1;36(Database issue):D83–7. doi: 10.1093/nar/gkm875 17981843 10.1093/nar/gkm875 PMC2238977

[40] Hirayama T, Tarusawa E, Yoshimura Y, Galjart N, Yagi T. CTCF is required for neural development and stochastic expression of clustered Pcdh genes in neurons. Cell Rep. 2012 8 30;2(2):345–57. doi: 10.1016/j.celrep.2012.06.014 22854024 10.1016/j.celrep.2012.06.014

[41] Fujita Y, Masuda K, Bando M, Nakato R, Katou Y, Tanaka T, et al Decreased cohesin in the brain leads to defective synapse development and anxiety-related behavior. J Exp Med. 2017 5 1;214(5):1431–1452. doi: 10.1084/jem.20161517 28408410 10.1084/jem.20161517 PMC5413336

[42] Stranger BE, Nica AC, Forrest MS, Dimas A, Bird CP, Beazley C, et al Population genomics of human gene expression. Nat Genet. 2007 10;39(10):1217–24. doi: 10.1038/ng2142 17873874 10.1038/ng2142 PMC2683249

[43] Schadt EE, Molony C, Chudin E, Hao K, Yang X, Lum PY, et al Mapping the genetic architecture of gene expression in human liver. PLoS Biol. 2008 5 6;6(5):e107 doi: 10.1371/journal.pbio.0060107 18462017 10.1371/journal.pbio.0060107 PMC2365981

[44] Ingason A, Rujescu D, Cichon S, Sigurdsson E, Sigmundsson T, Pietiläinen OP, et al Copy number variations of chromosome 16p13.1 region associated with schizophrenia. Mol Psychiatry. 2011 1;16(1):17–25. doi: 10.1038/mp.2009.101 19786961 10.1038/mp.2009.101 PMC3330746

