# Supplementary material for: Association studies of WD repeat domain 3 and chitobiosyldiphosphodolichol beta-mannosyltransferase genes with schizophrenia in a Japanese population
Source: PLoS One. 2018 Jan 8;13(1):e0190991. doi: 10.1371/journal.pone.0190991 (PMC5757935; doi:10.1371/journal.pone.0190991)
Supplement: S1 Table — N: number of subjects, HWE: Hardy-Weinberg equilibrium, MAF: minor allele frequency, FDR: the false discovery rate using the Benjamini-Hochberg procedure, OR: odds ratio, 95% CI: 95% confidence interval, CON: control, SCZ: schizophrenia. (PDF) [file pone.0190991.s001.pdf]

S1 Table. Stratification analysis of onset-age groups on *WDR3* and *ALG1* genes in schizophrenia and controls from Japanese population

a) <18 years old, or 18 years old and greater

WDR3

SNP ID rs number	Affection	Age at onset	N	HWE *P*	Allelic count		MAF	Allelic *P*	(FDR *P*)	OR (95% CI)	Genotypic count			Genotypic *P*	(FDR *P*)
					C	T					CC	CT	TT		
W1	CON		2,168	0.086	3,400	936	0.216				1,319	762	87		
rs1812607	SCZ	Under 17	264	0.726	408	120	0.227	0.539	(0.752)	1.068 (0.861-1.326)	156	96	12	0.787	(0.827)
		Over 18	1,424	0.194	2,211	637	0.224	0.448	(0.616)	1.047 (0.934-1.173)	867	477	80	0.067	(0.198)
					A	T					AA	AT	TT		
W2	CON		2,168	0.066	3,402	934	0.215				1,320	762	86		
rs965361	SCZ	Under 17	264	0.482	409	119	0.225	0.615	(0.752)	1.060 (0.853-1.316)	156	97	11	0.827	(0.827)
		Over 18	1,426	0.195	2,214	638	0.224	0.414	(0.616)	1.050 (0.937-1.176)	868	478	80	0.060	(0.198)
					C	T					CC	CT	TT		
W4	CON		2,170	0.914	3,851	489	0.113				1,709	433	28		
rs319471	SCZ	Under 17	264	**0.048**	480	48	0.091	0.141	(0.388)	0.788 (0.577-1.075)	221	38	5	0.062	(0.341)
		Over 18	1,425	0.667	2,558	292	0.102	0.175	(0.385)	0.899 (0.771-1.048)	1,146	266	13	0.356	(0.559)
					T	A					TT	TA	AA		
W5	CON		2,168	0.122	2,189	2,147	0.495				534	1,121	513		
rs379058	SCZ	Under 17	264	0.622	259	269	0.509	0.549	(0.752)	1.059 (0.884-1.269)	61	137	66	0.823	(0.827)
		Over 18	1,426	0.671	1,479	1,373	0.481	0.257	(0.471)	0.947 (0.861-1.040)	379	721	326	0.419	(0.576)
					C	T					CC	CT	TT		
W6	CON		2,169	0.428	3,548	790	0.182				1,445	658	66		
rs3754127	SCZ	Under 17	263	0.855	414	112	0.213	0.096	(0.352)	1.215 (0.973-1.518)	162	90	11	0.196	(0.539)
		Over 18	1,426	0.659	2,329	523	0.183	0.901	(0.939)	1.009 (0.893-1.140)	948	433	45	0.977	(0.996)
					A	C					AA	AC	CC		
W7	CON		2,169	0.530	4,180	158	0.036				2,015	150	4		
rs17037749	SCZ	Under 17	264	1.000	507	21	0.040	0.713	(0.784)	1.096 (0.689-1.744)	243	21	0	0.700	(0.827)
		Over 18	1,426	0.081	2,754	98	0.034	0.696	(0.851)	0.941 (0.728-1.217)	1,332	90	4	0.621	(0.759)
					G	C					GG	GC	CC		
W8	CON		2,169	0.088	3,575	763	0.176				1,485	605	79		
rs1321663	SCZ	Under 17	264	0.822	442	86	0.163	0.504	(0.752)	0.912 (0.714-1.163)	184	74	6	0.580	(0.827)
		Over 18	1,425	0.312	2,292	558	0.196	**0.034**	(0.374)	**1.141 (1.011-1.288)**	915	462	48	**0.015**	(0.165)
					T	C					TT	TC	CC		
W10	CON		2,167	0.697	2,328	2,006	0.463				630	1,068	469		
rs1321666	SCZ	Under 17	263	1.000	273	253	0.481	0.432	(0.752)	1.075 (0.897-1.289)	71	131	61	0.727	(0.827)
		Over 18	1,423	0.750	1,471	1,375	0.483	0.095	(0.385)	1.085 (0.987-1.193)	377	717	329	0.212	(0.466)
					G	C					GG	GC	CC		
W12	CON		2,169	0.208	3,684	654	0.151				1,572	540	57		
rs10802003	SCZ	Under 17	264	0.146	463	65	0.123	0.092	(0.352)	0.791 (0.602-1.039)	200	63	1	**0.041**	(0.341)
		Over 18	1,426	0.247	2,385	467	0.164	0.144	(0.385)	1.103 (0.969-1.255)	991	403	32	0.072	(0.198)
					C	T					CC	CT	TT		
W13	CON		2,170	0.884	3,563	777	0.179				1,461	641	68		
rs10754369	SCZ	Under 17	263	0.806	449	77	0.146	0.069	(0.352)	0.786 (0.610-1.014)	192	65	6	0.191	(0.539)
		Over 18	1,426	0.933	2,298	554	0.194	0.107	(0.385)	1.105 (0.980-1.248)	925	448	53	0.262	(0.480)
					C	T					CC	CT	TT		
W15	CON		2,169	0.827	3,859	479	0.110				1,715	429	25		
rs3753261	SCZ	Under 17	264	0.548	468	60	0.114	0.826	(0.826)	1.033 (0.777-1.374)	206	56	2	0.796	(0.827)
		Over 18	1,426	1.000	2,535	317	0.111	0.939	(0.939)	1.007 (0.867-1.171)	1,126	283	17	0.996	(0.996)

N: number of subjects, HWE: Hardy-Weinberg equilibrium, MAF: minor allele frequency, FDR: the false discovery rate using the Benjamini-Hochberg procedure, OR: odds ratio, 95% CI: 95% confidence interval, CON: control, SCZ: schizophrenia

ALG1

SNP ID rs number	Affection	Age at onset	N	HWE *P*	Allelic count		MAF	Allelic *P*	(FDR *P*)	OR (95% CI)	Genotypic count			Genotypic *P*	(FDR *P*)
					C	G					CC	GC	GG		
A1	CON		2,169	0.211	2,987	1,351	0.311				1,041	905	223		
rs8053916	SCZ	Under 17	264	0.881	377	151	0.286	0.251	(0.661)	0.886 (0.726-1.081)	135	107	22	0.517	(0.898)
		Over 18	1,425	0.704	2,003	847	0.297	0.209	(0.321)	0.935 (0.844-1.036)	707	589	129	0.404	(0.544)
					C	T					CC	CT	TT		
A2	CON		2,170	0.656	3,211	1,129	0.260				1,192	827	151		
rs9924614	SCZ	Under 17	264	0.876	386	142	0.269	0.675	(0.844)	1.046 (0.853-1.283)	140	106	18	0.808	(0.898)
		Over 18	1,426	0.252	2,153	699	0.245	0.158	(0.321)	0.923 (0.828-1.030)	821	511	94	0.296	(0.544)
					C	T					CC	TC	TT		
A3	CON		2,161	0.769	3,550	772	0.179				1,460	630	71		
rs9932909	SCZ	Under 17	262	0.179	438	86	0.164	0.431	(0.661)	0.903 (0.708-1.152)	186	66	10	0.366	(0.898)
		Over 18	1,419	0.581	2,341	497	0.175	0.728	(0.809)	0.976 (0.862-1.105)	962	417	40	0.744	(0.744)
					C	T					CC	TC	TT		
A4	CON		2,165	0.543	3,337	993	0.229				1,291	755	119		
rs3760030	SCZ	Under 17	263	0.245	397	129	0.245	0.412	(0.661)	1.092 (0.884-1.348)	146	105	12	0.271	(0.898)
		Over 18	1,426	0.296	2,197	655	0.230	0.977	(0.977)	1.002 (0.895-1.121)	839	519	68	0.470	(0.544)
					C	T					CC	TC	TT		
A5	CON		2,166	0.686	3,647	685	0.158				1,532	583	51		
rs3760029	SCZ	Under 17	263	0.594	455	71	0.135	0.181	(0.661)	0.831 (0.639-1.081)	198	59	6	0.285	(0.898)
		Over 18	1,424	0.672	2,432	416	0.146	0.170	(0.321)	0.911 (0.798-1.039)	1,036	360	28	0.384	(0.544)
					T	C					TT	CT	CC		
A6	CON		2,147	0.415	3,701	593	0.138				1,590	521	36		
rs3760027	SCZ	Under 17	263	0.811	447	79	0.150	0.463	(0.661)	1.103 (0.855-1.423)	189	69	5	0.700	(0.898)
		Over 18	1,415	0.831	2,418	412	0.146	0.385	(0.481)	1.063 (0.928-1.218)	1,034	350	31	0.490	(0.544)
					G	C					GG	CG	CC		
A7	CON		2,166	0.822	2,630	1,702	0.393				801	1,028	337		
rs8045294	SCZ	Under 17	262	0.362	322	202	0.385	0.776	(0.862)	0.969 (0.805-1.168)	95	132	35	0.574	(0.898)
		Over 18	1,423	0.195	1,769	1,077	0.378	0.225	(0.321)	0.941 (0.854-1.037)	538	693	192	0.232	(0.544)
					C	G					CC	GC	GG		
A8	CON		2,166	0.636	2,197	2,135	0.493				551	1,095	520		
rs8045473	SCZ	Under 17	262	0.806	265	259	0.494	0.963	(0.963)	1.006 (0.839-1.206)	68	129	65	0.915	(0.915)
		Over 18	1,424	0.634	1,397	1,451	0.509	0.169	(0.321)	1.069 (0.972-1.175)	338	721	365	0.383	(0.544)
					C	T					CC	TC	TT		
A9	CON		2,160	0.856	3,728	592	0.137				1,607	514	39		
rs7195893	SCZ	Under 17	263	1.000	461	65	0.124	0.419	(0.661)	0.888 (0.675-1.168)	202	57	4	0.757	(0.898)
		Over 18	1,421	0.708	2,499	343	0.121	**0.045**	(0.321)	**0.864 (0.750-0.997)**	1,100	299	22	0.121	(0.544)
					C	G					CC	CG	GG		
A10	CON		2,170	0.563	3,552	788	0.182				1,449	654	67		
rs9673733	SCZ	Under 17	264	0.486	445	83	0.157	0.186	(0.661)	0.841 (0.657-1.076)	189	67	8	0.262	(0.898)
		Over 18	1,426	0.564	2,381	471	0.165	0.076	(0.321)	0.892 (0.787-1.011)	997	387	42	0.136	(0.544)

N: number of subjects, HWE: Hardy-Weinberg equilibrium, MAF: minor allele frequency, FDR: the false discovery rate using the Benjamini-Hochberg procedure, OR: odds ratio, 95% CI: 95% confidence interval, CON: control, SCZ: schizophrenia

b) <16 years old, 16-25 years old, 26-35 years old, or 36 years old and greater

WDR3

SNP ID rs number	Affection	Age at onset	N	HWE *P*	Allelic count		MAF	Allelic *P*	(FDR *P*)	OR (95% CI)	Genotypic count			Genotypic *P*	(FDR *P*)
					C	T					CC	CT	TT		
W1	CON		2,168	0.086	3,400	936	0.216				1,319	762	87		
rs1812607	SCZ	Under 15	107	0.617	159	55	0.257	0.174	(0.561)	1.257 (0.917-1.722)	60	39	8	0.183	(0.746)
		16-25	917	0.351	1,414	420	0.229	0.253	(0.696)	1.079 (0.947-1.230)	550	314	53	0.102	(0.326)
		26-35	460	0.774	733	187	0.203	0.425	(0.555)	0.927 (0.777-1.105)	293	147	20	0.411	(0.565)
		Over 36	204	1.000	313	95	0.233	0.415	(0.761)	1.103 (0.867-1.403)	120	73	11	0.549	(0.834)
					A	T					AA	AT	TT		
W2	CON		2,168	0.066	3,402	934	0.215				1,320	762	86		
rs965361	SCZ	Under 15	107	1.000	160	54	0.252	0.203	(0.561)	1.229 (0.896-1.688)	60	40	7	0.302	(0.746)
		16-25	918	0.400	1,415	421	0.229	0.239	(0.696)	1.084 (0.951-1.235)	550	315	53	0.091	(0.326)
		26-35	461	0.773	735	187	0.203	0.426	(0.555)	0.927 (0.777-1.105)	294	147	20	0.397	(0.565)
		Over 36	204	1.000	313	95	0.233	0.414	(0.761)	1.106 (0.869-1.406)	120	73	11	0.538	(0.834)
					C	T					CC	CT	TT		
W4	CON		2,170	0.914	3,851	489	0.113				1,709	433	28		
rs319471	SCZ	Under 15	107	0.410	199	15	0.070	0.057	(0.561)	0.594 (0.348-1.012)	93	13	1	0.113	(0.746)
		16-25	917	0.243	1,629	205	0.112	0.965	(1)	0.991 (0.834-1.178)	727	175	15	0.642	(0.936)
		26-35	461	0.565	839	83	0.090	**0.048**	(0.223)	**0.779 (0.610-0.995)**	380	79	2	0.111	(0.442)
		Over 36	204	0.381	371	37	0.091	0.187	(0.761)	0.785 (0.553-1.115)	167	37	0	0.235	(0.834)
					T	A					TT	TA	AA		
W5	CON		2,168	0.122	2,189	2,147	0.495				534	1,121	513		
rs379058	SCZ	Under 15	107	0.847	106	108	0.505	0.834	(0.834)	1.039 (0.789-1.367)	27	52	28	0.758	(0.829)
		16-25	918	0.129	950	886	0.483	0.373	(0.821)	0.951 (0.853-1.061)	234	482	202	0.598	(0.936)
		26-35	461	0.779	488	434	0.471	0.180	(0.330)	0.907 (0.786-1.045)	131	226	104	0.241	(0.442)
		Over 36	204	0.578	194	214	0.525	0.277	(0.761)	1.125 (0.9178-1.378)	48	98	58	0.315	(0.834)
					C	T					CC	CT	TT		
W6	CON		2,169	0.428	3,548	790	0.182				1,445	658	66		
rs3754127	SCZ	Under 15	106	1.000	166	46	0.217	0.204	(0.561)	1.245 (0.890-1.741)	65	36	5	0.339	(0.746)
		16-25	918	0.827	1,496	340	0.185	0.773	(1)	1.021 (0.887-1.175)	608	280	30	0.923	(0.978)
		26-35	461	0.653	744	178	0.193	0.454	(0.555)	1.074 (0.897-1.287)	298	148	15	0.713	(0.871)
		Over 36	204	1.000	337	71	0.174	0.737	(0.772)	0.946 (0.724-1.236)	139	59	6	0.917	(0.917)
					A	C					AA	AC	CC		
W7	CON		2,169	0.530	4,180	158	0.036				2,015	150	4		
rs17037749	SCZ	Under 15	107	1.000	209	5	0.023	0.448	(0.677)	0.633 (0.257-1.558)	102	5	0	0.631	(0.829)
		16-25	918	1.000	1,770	66	0.036	1.000	(1)	0.987 (0.736-1.322)	853	64	1	0.978	(0.978)
		26-35	461	0.407	891	31	0.034	0.770	(0.847)	0.921 (0.622-1.362)	431	29	1	0.811	(0.892)
		Over 36	204	**0.038**	391	17	0.042	0.582	(0.772)	1.150 (0.690-1.917)	189	13	2	0.139	(0.834)
					G	C					GG	GC	CC		
W8	CON		2,169	0.088	3,575	763	0.176				1,485	605	79		
rs1321663	SCZ	Under 15	107	0.762	171	43	0.201	0.359	(0.658)	1.178 (0.836-1.661)	69	33	5	0.571	(0.829)
		16-25	917	0.110	1,481	353	0.192	0.129	(0.696)	1.117 (0.971-1.285)	590	301	26	**0.017**	(0.187)
		26-35	461	0.884	741	181	0.196	0.143	(0.315)	1.144 (0.956-1.371)	297	147	17	0.213	(0.442)
		Over 36	204	0.799	341	67	0.164	0.586	(0.772)	0.921 (0.700-1.210)	143	55	6	0.875	(0.917)
					T	C					TT	TC	CC		
W10	CON		2,167	0.697	2,328	2,006	0.463				630	1,068	469		
rs1321666	SCZ	Under 15	107	0.121	111	103	0.481	0.623	(0.685)	1.077 (0.818-1.417)	33	45	29	0.264	(0.746)
		16-25	915	0.290	940	890	0.486	0.094	(0.696)	1.099 (0.985-1.226)	233	474	208	0.124	(0.326)
		26-35	460	1.000	465	455	0.495	0.081	(0.223)	1.136 (0.985-1.309)	117	231	112	0.210	(0.442)
		Over 36	204	0.776	228	180	0.441	0.406	(0.761)	0.916 (0.747-1.124)	65	98	41	0.683	(0.834)
					G	C					GG	GC	CC		
W12	CON		2,169	0.208	3,684	654	0.151				1,572	540	57		
rs10802003	SCZ	Under 15	107	0.690	186	28	0.131	0.492	(0.677)	0.848 (0.565-1.273)	80	26	1	0.692	(0.829)
		16-25	918	0.164	1,553	283	0.154	0.756	(1)	1.026 (0.882-1.194)	651	251	16	0.148	(0.326)
		26-35	461	0.872	760	162	0.176	0.064	(0.223)	1.201 (0.994-1.450)	312	136	13	0.108	(0.442)
		Over 36	204	0.775	349	59	0.145	0.772	(0.772)	0.952 (0.714-1.270)	148	53	3	0.668	(0.834)
					C	T					CC	CT	TT		
W13	CON		2,170	0.884	3,563	777	0.179				1,461	641	68		
rs10754369	SCZ	Under 15	106	1.000	180	32	0.151	0.357	(0.658)	0.815 (0.555-1.197)	76	28	2	0.666	(0.829)
		16-25	918	0.376	1,503	333	0.181	0.828	(1)	1.016 (0.882-1.171)	619	265	34	0.681	(0.936)
		26-35	461	0.776	733	189	0.205	0.068	(0.223)	1.182 (0.990-1.412)	290	153	18	0.161	(0.442)
		Over 36	204	0.368	331	77	0.189	0.637	(0.772)	1.067 (0.823-1.383)	132	67	5	0.573	(0.834)
					C	T					CC	CT	TT		
W15	CON		2,169	0.827	3,859	479	0.110				1,715	429	25		
rs3753261	SCZ	Under 15	107	1.000	188	26	0.121	0.578	(0.685)	1.114 (0.731-1.697)	82	24	1	0.829	(0.829)
		16-25	918	0.730	1,640	196	0.107	0.688	(1)	0.963 (0.808-1.148)	731	178	9	0.910	(0.978)
		26-35	461	0.816	819	103	0.112	0.908	(0.908)	1.013 (0.808-1.270)	364	91	6	0.934	(0.934)
		Over 36	204	1.000	356	52	0.127	0.286	(0.761)	1.177 (0.867-1.598)	155	46	3	0.505	(0.834)

N: number of subjects, HWE: Hardy-Weinberg equilibrium, MAF: minor allele frequency, FDR: the false discovery rate using the Benjamini-Hochberg procedure, OR: odds ratio, 95% CI: 95% confidence interval, CON: control, SCZ: schizophrenia

ALG1

SNP ID rs number	Affection	Age at onset	N	HWE *P*	Allelic count		MAF	Allelic *P*	(FDR *P*)	OR (95% CI)	Genotypic count			Genotypic *P*	(FDR *P*)
					C	G					CC	GC	GG		
A1	CON		2,169	0.211	2,987	1,351	0.311				1,041	905	223		
rs8053916	SCZ	Under 15	107	0.094	154	60	0.280	0.364	(0.607)	0.861 (0.635-1.169)	59	36	12	0.235	(0.708)
		16-25	918	0.938	1,281	555	0.302	0.488	(0.697)	0.958 (0.851-1.078)	446	389	83	0.585	(0.731)
		26-35	461	0.510	643	279	0.303	0.610	(0.871)	0.959 (0.822-1.120)	221	201	39	0.465	(0.884)
		Over 36	203	0.196	302	104	0.256	**0.021**	(0.210)	**0.761 (0.604-0.960)**	116	70	17	**0.048**	(0.242)
					C	T					CC	CT	TT		
A2	CON		2,170	0.656	3,211	1,129	0.260				1,192	827	151		
rs9924614	SCZ	Under 15	107	1.000	155	59	0.276	0.632	(0.702)	1.083 (0.796-1.472)	56	43	8	0.846	(0.846)
		16-25	918	0.240	1,394	442	0.241	0.110	(0.443)	0.902 (0.795-1.024)	536	322	60	0.210	(0.568)
		26-35	461	1.000	691	231	0.251	0.562	(0.871)	0.951 (0.807-1.120)	259	173	29	0.846	(0.910)
		Over 36	204	0.859	299	109	0.267	0.768	(0.863)	1.037 (0.824-1.304)	110	79	15	0.919	(0.919)
					C	T					CC	TC	TT		
A3	CON		2,161	0.769	3,550	772	0.179				1,460	630	71		
rs9932909	SCZ	Under 15	106	0.057	180	32	0.151	0.357	(0.607)	0.818 (0.557-1.200)	79	22	5	0.129	(0.645)
		16-25	914	0.567	1,508	320	0.175	0.770	(0.791)	0.976 (0.845-1.126)	619	270	25	0.742	(0.824)
		26-35	458	0.418	756	160	0.175	0.812	(0.902)	0.973 (0.807-1.174)	309	138	11	0.619	(0.884)
		Over 36	203	0.219	335	71	0.175	0.892	(0.892)	0.9746 (0.746-1.274)	141	53	9	0.457	(0.584)
					C	T					CC	TC	TT		
A4	CON		2,165	0.543	3,337	993	0.229				1,291	755	119		
rs3760030	SCZ	Under 15	106	0.395	167	45	0.212	0.616	(0.702)	0.906 (0.647-1.268)	64	39	3	0.561	(0.775)
		16-25	918	1.000	1,409	427	0.233	0.791	(0.791)	1.018 (0.895-1.159)	540	329	49	0.877	(0.877)
		26-35	461	0.130	698	224	0.243	0.390	(0.780)	1.078 (0.913-1.274)	258	182	21	0.161	(0.537)
		Over 36	204	0.408	320	88	0.216	0.579	(0.863)	0.924 (0.722-1.182)	123	74	7	0.467	(0.584)
					C	T					CC	TC	TT		
A5	CON		2,166	0.686	3,647	685	0.158				1,532	583	51		
rs3760029	SCZ	Under 15	106	0.079	184	28	0.132	0.335	(0.607)	0.810 (0.540-1.216)	82	20	4	0.111	(0.645)
		16-25	917	0.894	1,569	265	0.144	0.177	(0.443)	0.899 (0.771-1.049)	670	229	18	0.408	(0.680)
		26-35	460	0.342	788	132	0.143	0.293	(0.780)	0.892 (0.729-1.091)	340	108	12	0.296	(0.740)
		Over 36	204	**0.006**	346	62	0.152	0.777	(0.863)	0.954 (0.719-1.265)	142	62	0	**0.031**	(0.242)
					T	C					TT	CT	CC		
A6	CON		2,147	0.415	3,701	593	0.138				1,590	521	36		
rs3760027	SCZ	Under 15	106	1.000	184	28	0.132	0.919	(0.919)	0.950 (0.632-1.427)	80	24	2	0.841	(0.846)
		16-25	912	0.496	1,565	259	0.142	0.687	(0.791)	1.033 (0.882-1.209)	674	217	21	0.482	(0.689)
		26-35	456	0.404	758	154	0.169	**0.019**	(0.190)	**1.268 (1.045-1.539)**	312	134	10	**0.045**	(0.450)
		Over 36	204	1.000	358	50	0.123	0.408	(0.863)	0.872 (0.640-1.186)	157	44	3	0.684	(0.760)
					G	C					GG	CG	CC		
A7	CON		2,166	0.822	2,630	1,702	0.393				801	1,028	337		
rs8045294	SCZ	Under 15	106	0.675	137	75	0.354	0.280	(0.607)	0.846 (0.634-1.128)	43	51	12	0.475	(0.775)
		16-25	916	0.440	1,139	693	0.378	0.290	(0.483)	0.940 (0.840-1.052)	348	443	125	0.398	(0.680)
		26-35	459	0.769	557	361	0.393	1.000	(1)	1.001 (0.866-1.159)	167	223	69	0.910	(0.910)
		Over 36	204	0.070	258	150	0.368	0.340	(0.863)	0.898 (0.728-1.109)	75	108	21	0.095	(0.269)
					C	G					CC	GC	GG		
A8	CON		2,166	0.636	2,197	2,135	0.493				551	1,095	520		
rs8045473	SCZ	Under 15	107	0.563	101	113	0.528	0.327	(0.607)	1.151 (0.875-1.516)	22	57	28	0.524	(0.775)
		16-25	915	0.261	898	932	0.509	0.242	(0.483)	1.068 (0.957-1.191)	229	440	246	0.227	(0.568)
		26-35	460	0.401	462	458	0.498	0.799	(0.902)	1.020 (0.885-1.176)	111	240	109	0.801	(0.910)
		Over 36	204	0.161	201	207	0.507	0.605	(0.863)	1.060 (0.865-1.298)	44	113	47	0.374	(0.584)
					C	T					CC	TC	TT		
A9	CON		2,160	0.856	3,728	592	0.137				1,607	514	39		
rs7195893	SCZ	Under 15	107	0.631	189	25	0.117	0.474	(0.677)	0.833 (0.544-1.275)	84	21	2	0.620	(0.775)
		16-25	916	0.338	1,616	216	0.118	**0.043**	(0.430)	**0.842 (0.713-0.994)**	716	184	16	0.075	(0.568)
		26-35	458	0.673	801	115	0.126	0.366	(0.780)	0.904 (0.730-1.120)	351	99	8	0.604	(0.884)
		Over 36	203	0.051	354	52	0.128	0.651	(0.863)	0.925 (0.683-1.253)	151	52	0	0.108	(0.269)
					C	G					CC	CG	GG		
A10	CON		2,170	0.563	3,552	788	0.182				1,449	654	67		
rs9673733	SCZ	Under 15	107	0.268	181	33	0.154	0.362	(0.607)	0.822 (0.563-1.200)	78	25	4	0.283	(0.708)
		16-25	918	0.475	1,532	304	0.166	0.135	(0.443)	0.895 (0.774-1.034)	642	248	28	0.213	(0.568)
		26-35	461	0.229	772	150	0.163	0.185	(0.780)	0.876 (0.723-1.060)	327	118	16	0.141	(0.537)
		Over 36	204	0.121	341	67	0.164	0.419	(0.863)	0.886 (0.674-1.164)	139	63	2	0.241	(0.482)

N: number of subjects, HWE: Hardy-Weinberg equilibrium, MAF: minor allele frequency, FDR: the false discovery rate using the Benjamini-Hochberg procedure, OR: odds ratio, 95% CI: 95% confidence interval, CON: control, SCZ: schizophrenia
