# Supplementary material for: Association studies of WD repeat domain 3 and chitobiosyldiphosphodolichol beta-mannosyltransferase genes with schizophrenia in a Japanese population
Source: PLoS One. 2018 Jan 8;13(1):e0190991. doi: 10.1371/journal.pone.0190991 (PMC5757935; doi:10.1371/journal.pone.0190991)
Supplement: S2 Table — N: number of subjects, HWE: Hardy-Weinberg equilibrium, MAF: minor allele frequency, FDR: the false discovery rate using the Benjamini-Hochberg procedure, OR: odds ratio, 95% CI: 95% confidence interval, CON: control, SCZ: schizophrenia. (PDF) [file pone.0190991.s002.pdf]

S2 Table. Stratification analysis of onset-age groups by sex on *WDR3* and *ALG1* genes in schizophrenia and controls from Japanese population

a) Male

WDR3

SNP ID rs number	Affection	Age at onset	N	HWE *P*	Allelic count		MAF	Allelic *P*	(FDR *P*)	OR (95% CI)	Genotypic count			Genotypic *P*	(FDR *P*)
					C	T					CC	CT	TT		
W1	CON		887	0.211	1,374	400	0.225				525	324	38		
rs1812607	SCZ	Under 15	50	0.702	77	23	0.230	0.902	(1)	1.026 (0.636-1.656)	30	17	3	0.709	(0.959)
		16-25	519	0.534	800	238	0.229	0.816	(0.920)	1.022 (0.851-1.227)	311	178	30	0.364	(0.819)
		26-35	252	0.831	415	89	0.177	**0.019**	(0.105)	**0.737 (0.571-0.950)**	170	75	7	0.054	(0.297)
		Over 36	94	1.000	144	44	0.234	0.784	(0.958)	1.050 (0.736-1.498)	55	34	5	0.842	(0.926)
					A	T					AA	AT	TT		
W2	CON		887	0.211	1,374	400	0.225				525	324	38		
rs965361	SCZ	Under 15	50	1.000	78	22	0.220	1.000	(1)	0.969 (0.596-1.575)	30	18	2	1.000	(1)
		16-25	519	0.534	800	238	0.229	0.816	(0.920)	1.022 (0.851-1.227)	311	178	30	0.364	(0.819)
		26-35	252	0.831	415	89	0.177	**0.019**	(0.105)	**0.737 (0.571-0.950)**	170	75	7	0.054	(0.297)
		Over 36	94	1.000	144	44	0.234	0.784	(0.958)	1.050 (0.736-1.498)	55	34	5	0.842	(0.926)
					C	T					CC	CT	TT		
W4	CON		889	0.110	1,588	190	0.107				714	160	15		
rs319471	SCZ	Under 15	50	0.326	91	9	0.090	0.738	(1)	0.827 (0.410-1.667)	42	7	1	0.611	(0.959)
		16-25	518	**0.010**	913	123	0.119	0.351	(0.920)	1.126 (0.885-1.433)	409	95	14	0.420	(0.819)
		26-35	252	0.486	453	51	0.101	0.743	(0.817)	0.941 (0.679-1.304)	202	49	1	0.306	(0.481)
		Over 36	94	0.591	168	20	0.106	1.000	(1)	0.995 (0.611-1.620)	74	20	0	0.430	(0.926)
					T	A					TT	TA	AA		
W5	CON		888	0.591	879	879	0.500				222	453	213		
rs379058	SCZ	Under 15	50	0.166	54	46	0.460	0.538	(1)	0.869 (0.580-1.302)	17	20	13	0.249	(0.959)
		16-25	519	0.725	544	494	0.476	0.348	(0.920)	0.927 (0.795-1.080)	140	264	115	0.617	(0.819)
		26-35	252	0.448	275	229	0.454	0.118	(0.325)	0.850 (0.697-1.036)	78	119	55	0.173	(0.481)
		Over 36	94	0.152	92	96	0.511	0.702	(0.958)	1.065 (0.788-1.438)	26	40	28	0.258	(0.926)
					C	T					CC	CT	TT		
W6	CON		888	0.653	1,451	325	0.183				590	271	27		
rs3754127	SCZ	Under 15	49	1.000	76	22	0.224	0.288	(0.839)	1.292 (0.792-2.109)	29	18	2	0.457	(0.959)
		16-25	519	0.554	850	188	0.181	0.920	(0.920)	0.988 (0.810-1.204)	350	150	19	0.694	(0.819)
		26-35	252	0.700	402	102	0.202	0.332	(0.609)	1.133 (0.884-1.452)	159	84	9	0.570	(0.697)
		Over 36	94	0.725	156	32	0.170	0.765	(0.958)	0.916 (0.615-1.365)	65	26	3	0.825	(0.926)
					A	C					AA	AC	CC		
W7	CON		889	1.000	1,716	62	0.035				828	60	1		
rs17037749	SCZ	Under 15	50	1.000	97	3	0.030	0.288	(0.839)	0.856 (0.264-2.776)	47	3	0	1.000	(1)
		16-25	519	1.000	1,007	31	0.030	0.513	(0.920)	0.852 (0.550-1.320)	488	31	0	0.781	(0.819)
		26-35	252	0.409	481	23	0.046	0.286	(0.609)	1.323 (0.812-2.158)	230	21	1	0.288	(0.481)
		Over 36	94	1.000	184	4	0.021	0.400	(0.958)	0.602 (0.216-1.673)	90	4	0	0.555	(0.926)
					G	C					GG	GC	CC		
W8	CON		889	0.911	1,449	329	0.185				591	267	31		
rs1321663	SCZ	Under 15	50	0.702	77	23	0.230	0.291	(0.839)	1.316 (0.813-2.128)	30	17	3	0.404	(0.959)
		16-25	519	0.478	839	199	0.192	0.689	(0.920)	1.045 (0.859-1.270)	336	167	16	0.671	(0.819)
		26-35	252	0.838	409	95	0.188	0.846	(0.846)	1.023 (0.794-1.318)	165	79	8	0.925	(0.925)
		Over 36	94	0.709	157	31	0.165	0.552	(0.958)	0.870 (0.581-1.302)	66	25	3	0.800	(0.926)
					T	C					TT	TC	CC		
W10	CON		888	0.590	947	829	0.467				248	451	189		
rs1321666	SCZ	Under 15	50	0.051	48	52	0.520	0.305	(0.839)	1.238 (0.827-1.852)	15	18	17	0.065	(0.715)
		16-25	516	0.428	539	493	0.478	0.583	(0.920)	1.045 (0.896-1.218)	136	267	113	0.819	(0.819)
		26-35	252	0.801	247	257	0.510	0.095	(0.325)	1.189 (0.975-1.449)	59	129	64	0.226	(0.481)
		Over 36	94	0.285	112	76	0.404	0.107	(0.958)	0.775 (0.571-1.052)	36	40	18	0.117	(0.926)
					G	C					GG	GC	CC		
W12	CON		889	0.799	1,503	275	0.155				636	231	22		
rs10802003	SCZ	Under 15	50	0.329	84	16	0.160	0.887	(1)	1.041 (0.601-1.804)	34	16	0	0.459	(0.959)
		16-25	519	0.330	870	168	0.162	0.629	(0.920)	1.055 (0.856-1.301)	361	148	10	0.513	(0.819)
		26-35	252	0.257	419	85	0.169	0.447	(0.702)	1.109 (0.850-1.447)	177	65	10	0.427	(0.587)
		Over 36	94	0.419	160	28	0.149	0.916	(1)	0.957 (0.627-1.458)	69	22	3	0.724	(0.926)
					C	T					CC	CT	TT		
W13	CON		889	0.114	1,456	322	0.181				589	278	22		
rs10754369	SCZ	Under 15	49	0.576	83	15	0.153	0.589	(1)	0.817 (0.465-1.435)	34	15	0	0.785	(0.959)
		16-25	519	0.886	842	196	0.189	0.615	(0.920)	1.053 (0.865-1.281)	342	158	19	0.440	(0.819)
		26-35	252	0.410	409	95	0.188	0.696	(0.817)	1.050 (0.815-1.354)	168	73	11	0.243	(0.481)
		Over 36	94	0.316	152	36	0.191	0.692	(0.958)	1.071 (0.730-1.570)	63	26	5	0.246	(0.926)
					C	T					CC	CT	TT		
W15	CON		889	0.328	1,571	207	0.116				697	177	15		
rs3753261	SCZ	Under 15	50	0.460	89	11	0.110	1.000	(1)	0.938 (0.493-1.784)	40	9	1	0.775	(0.959)
		16-25	519	0.661	920	118	0.114	0.855	(0.920)	0.973 (0.766-1.238)	406	108	5	0.539	(0.819)
		26-35	252	0.749	449	55	0.109	0.693	(0.817)	0.930 (0.678-1.274)	199	51	2	0.696	(0.766)
		Over 36	94	1.000	169	19	0.101	0.630	(0.958)	0.853 (0.520-1.401)	76	17	1	0.927	(0.927)

N: number of subjects, HWE: Hardy-Weinberg equilibrium, MAF: minor allele frequency, FDR: the false discovery rate using the Benjamini-Hochberg procedure, OR: odds ratio, 95% CI: 95% confidence interval, CON: control, SCZ: schizophrenia

ALG1

SNP ID rs number	Affection	Age at onset	N	HWE *P*	Allelic count		MAF	Allelic *P*	(FDR *P*)	OR (95% CI)	Genotypic count			Genotypic *P*	(FDR *P*)
					C	G					CC	GC	GG		
A1	CON		888	0.177	1,238	538	0.303				440	358	90		
rs8053916	SCZ	Under 15	50	0.099	77	23	0.230	0.144	(0.420)	0.687 (0.427-1.107)	32	13	5	0.101	(0.433)
		16-25	519	1.000	730	308	0.297	0.734	(0.734)	0.971 (0.821-1.148)	257	216	46	0.714	(0.882)
		26-35	252	0.759	359	145	0.288	0.545	(0.694)	0.929 (0.748-1.156)	129	101	22	0.799	(0.873)
		Over 36	94	0.194	136	52	0.277	0.504	(1)	0.880 (0.629-1.230)	52	32	10	0.499	(0.880)
					C	T					CC	CT	TT		
A2	CON		889	0.732	1,302	476	0.268				479	344	66		
rs9924614	SCZ	Under 15	50	1.000	70	30	0.300	0.488	(0.697)	1.172 (0.755-1.821)	24	22	4	0.653	(0.653)
		16-25	249	1.000	788	250	0.241	0.118	(0.730)	0.868 (0.727-1.036)	29	190	30	0.295	(0.882)
		26-35	252	1.000	386	118	0.234	0.135	(0.675)	0.836 (0.664-1.054)	148	90	14	0.345	(0.868)
		Over 36	94	0.626	132	56	0.298	0.388	(1)	1.160 (0.835-1.614)	45	42	7	0.495	(0.880)
					C	T					CC	TC	TT		
A3	CON		882	0.907	1,456	308	0.175				600	256	26		
rs9932909	SCZ	Under 15	49	0.064	83	15	0.153	0.681	(0.757)	0.854 (0.486-1.501)	37	9	3	0.130	(0.433)
		16-25	518	0.775	839	197	0.190	0.309	(0.730)	1.110 (0.911-1.353)	338	163	17	0.552	(0.882)
		26-35	252	1.000	425	79	0.157	0.383	(0.694)	0.879 (0.671-1.151)	179	67	6	0.685	(0.868)
		Over 36	93	0.055	156	30	0.161	0.760	(1)	0.909 (0.604-1.369)	68	20	5	0.146	(0.880)
					C	T					CC	TC	TT		
A4	CON		884	0.344	1,360	408	0.231				528	304	52		
rs3760030	SCZ	Under 15	49	0.424	77	21	0.214	0.805	(0.805)	0.909 (0.554-1.491)	29	19	1	0.570	(0.653)
		16-25	519	0.549	787	251	0.242	0.519	(0.730)	1.063 (0.888-1.273)	301	185	33	0.794	(0.882)
		26-35	252	0.739	377	127	0.252	0.341	(0.694)	1.123 (0.893-1.413)	142	93	17	0.590	(0.868)
		Over 36	94	0.773	144	44	0.234	0.928	(1)	1.019 (0.714-1.453)	54	36	4	0.734	(0.880)
					C	T					CC	TC	TT		
A5	CON		889	0.535	1,493	285	0.160				624	245	20		
rs3760029	SCZ	Under 15	49	0.399	88	10	0.102	0.153	(0.420)	0.595 (0.306-1.159)	40	8	1	0.198	(0.495)
		16-25	519	1.000	884	154	0.148	0.420	(0.730)	0.913 (0.738-1.129)	376	132	11	0.674	(0.882)
		26-35	252	0.441	431	73	0.145	0.445	(0.694)	0.887 (0.672-1.172)	186	59	7	0.381	(0.868)
		Over 36	94	0.116	158	30	0.160	1.000	(1)	0.995 (0.660-1.499)	64	30	0	0.310	(0.880)
					T	C					TT	CT	CC		
A6	CON		880	0.888	1,514	246	0.140				650	214	16		
rs3760027	SCZ	Under 15	48	1.000	81	15	0.156	0.650	(0.757)	1.143 (0.648-2.016)	34	13	1	0.648	(0.653)
		16-25	515	0.391	875	155	0.150	0.433	(0.730)	1.093 (0.879-1.360)	374	127	14	0.526	(0.882)
		26-35	246	0.649	409	83	0.169	0.112	(0.675)	1.253 (0.954-1.644)	171	67	8	0.205	(0.868)
		Over 36	94	0.684	162	26	0.138	1.000	(1)	0.988 (0.639-1.527)	70	22	2	0.880	(0.880)
					G	C					GG	CG	CC		
A7	CON		888	0.727	1,066	710	0.400				317	432	139		
rs8045294	SCZ	Under 15	49	1.000	66	32	0.327	0.168	(0.420)	0.728 (0.472-1.122)	22	22	5	0.400	(0.653)
		16-25	518	0.713	631	405	0.391	0.660	(0.733)	0.964 (0.824-1.127)	190	251	77	0.899	(0.899)
		26-35	251	0.293	300	202	0.402	0.918	(0.918)	1.011 (0.826-1.237)	94	112	45	0.477	(0.868)
		Over 36	94	0.519	113	75	0.399	1.000	(1)	0.997 (0.733-1.355)	32	49	13	0.834	(0.880)
					C	G					CC	GC	GG		
A8	CON		888	0.503	908	868	0.489				237	434	217		
rs8045473	SCZ	Under 15	50	0.782	46	54	0.540	0.355	(0.613)	1.228 (0.820-1.840)	11	24	15	0.616	(0.653)
		16-25	518	0.335	518	518	0.500	0.584	(0.730)	1.046 (0.897-1.219)	135	248	135	0.794	(0.882)
		26-35	252	1.000	252	252	0.500	0.686	(0.762)	1.046 (0.858-1.275)	63	126	63	0.873	(0.873)
		Over 36	94	1.000	92	96	0.511	0.592	(1)	1.092 (0.808-1.475)	22	48	24	0.803	(0.880)
					C	T					CC	TC	TT		
A9	CON		885	0.673	1,524	246	0.139				654	216	15		
rs7195893	SCZ	Under 15	50	0.392	90	10	0.100	0.368	(0.613)	0.688 (0.353-1.341)	41	8	1	0.370	(0.653)
		16-25	518	0.545	909	127	0.123	0.227	(0.730)	0.866 (0.688-1.089)	400	109	9	0.354	(0.882)
		26-35	250	0.764	440	60	0.120	0.299	(0.694)	0.845 (0.625-1.142)	194	52	4	0.483	(0.868)
		Over 36	93	0.352	164	22	0.118	0.502	(1)	0.831 (0.522-1.323)	71	22	0	0.609	(0.880)
					C	G					CC	CG	GG		
A10	CON		889	0.911	1,453	325	0.183				594	265	30		
rs9673733	SCZ	Under 15	50	0.126	88	12	0.120	0.140	(0.420)	0.610 (0.330-1.128)	40	8	2	0.085	(0.433)
		16-25	519	0.642	860	178	0.171	0.475	(0.730)	0.925 (0.757-1.132)	358	144	17	0.708	(0.882)
		26-35	252	0.502	418	86	0.171	0.555	(0.694)	0.920 (0.708-1.195)	175	68	9	0.694	(0.868)
		Over 36	94	0.452	158	30	0.160	0.486	(1)	0.849 (0.564-1.277)	65	28	1	0.582	(0.880)

N: number of subjects, HWE: Hardy-Weinberg equilibrium, MAF: minor allele frequency, FDR: the false discovery rate using the Benjamini-Hochberg procedure, OR: odds ratio, 95% CI: 95% confidence interval, CON: control, SCZ: schizophrenia

b) Female

WDR3

SNP ID rs number	Affection	Age at onset	N	HWE *P*	Allelic count		MAF	Allelic *P*	(FDR *P*)	OR (95% CI)	Genotypic count			Genotypic *P*	(FDR *P*)
					C	T					CC	CT	TT		
W1	CON		1,281	0.272	2,026	536	0.209				794	438	49		
rs1812607	SCZ	Under 15	57	0.746	82	32	0.281	0.079	(0.263)	1.475 (0.970-2.244)	30	22	5	0.098	(0.357)
		16-25	398	0.569	614	182	0.229	0.255	(0.765)	1.120 (0.926-1.356)	239	136	23	0.240	(0.688)
		26-35	208	0.566	318	98	0.236	0.221	(0.408)	1.165 (0.911-1.489)	123	72	13	0.245	(0.381)
		Over 36	110	1.000	169	51	0.232	0.439	(0.731)	1.141 (0.822-1.582)	65	39	6	0.598	(0.748)
					A	T					AA	AT	TT		
W2	CON		1,281	0.235	2,028	534	0.208				795	438	48		
rs965361	SCZ	Under 15	57	0.746	82	32	0.281	0.078	(0.263)	1.482 (0.974-2.254)	30	22	5	0.089	(0.357)
		16-25	399	0.572	615	183	0.229	0.216	(0.765)	1.130 (0.934-1.367)	239	137	23	0.207	(0.688)
		26-35	209	0.564	320	98	0.234	0.245	(0.408)	1.163 (0.910-1.487)	124	72	13	0.239	(0.381)
		Over 36	110	1.000	169	51	0.232	0.438	(0.731)	1.146 (0.826-1.590)	65	39	6	0.538	(0.748)
					C	T					CC	CT	TT		
W4	CON		1,281	0.278	2,263	299	0.117				995	273	13		
rs319471	SCZ	Under 15	57	1.000	108	6	0.053	**0.034**	(0.263)	**0.421 (0.183-0.965)**	51	6	0	0.107	(0.357)
		16-25	399	0.102	716	82	0.103	0.306	(0.765)	0.867 (0.669-1.122)	318	80	1	0.315	(0.688)
		26-35	209	1.000	386	32	0.077	**0.015**	(0.125)	**0.627 (0.429-0.918)**	178	30	1	**0.044**	(0.147)
		Over 36	110	1.000	203	17	0.077	0.077	(0.386)	0.634 (0.381-1.055)	93	17	0	0.233	(0.466)
					T	A					TT	TA	AA		
W5	CON		1,280	0.131	1,292	1,268	0.495				312	668	300		
rs379058	SCZ	Under 15	57	0.426	52	62	0.544	0.339	(0.571)	1.215 (0.834-1.770)	10	32	15	0.486	(0.670)
		16-25	399	0.072	406	392	0.491	0.871	(0.871)	0.984 (0.839-1.153)	94	218	87	0.690	(0.767)
		26-35	209	0.783	213	205	0.490	0.874	(0.971)	0.981 (0.797-1.206)	53	107	49	0.945	(0.989)
		Over 36	110	0.571	102	118	0.536	0.261	(0.653)	1.179 (0.894-1.554)	22	58	30	0.488	(0.748)
					C	T					CC	CT	TT		
W6	CON		1,281	0.638	2,097	465	0.181				855	387	39		
rs3754127	SCZ	Under 15	57	0.694	90	24	0.211	0.457	(0.571)	1.203 (0.758-1.908)	36	18	3	0.536	(0.670)
		16-25	399	0.330	646	152	0.190	0.565	(0.807)	1.061 (0.866-1.300)	258	130	11	0.679	(0.767)
		26-35	209	0.818	342	76	0.182	1.000	(1)	1.002 (0.767-1.310)	139	64	6	0.989	(0.989)
		Over 36	110	1.000	181	39	0.177	0.928	(0.928)	0.972 (0.678-1.393)	74	33	3	1.000	(1)
					A	C					AA	AC	CC		
W7	CON		1,280	0.419	2,464	96	0.038				1,187	90	3		
rs17037749	SCZ	Under 15	57	1.000	112	2	0.018	0.439	(0.571)	0.458 (0.112-1.883)	55	2	0	0.496	(0.670)
		16-25	399	0.541	763	35	0.044	0.404	(0.807)	1.177 (0.793-1.748)	365	33	1	0.623	(0.767)
		26-35	209	1.000	410	8	0.019	0.061	(0.168)	0.501 (0.242-1.038)	201	8	0	0.186	(0.381)
		Over 36	110	**0.040**	207	13	0.059	0.144	(0.478)	1.612 (0.888-2.926)	99	9	2	0.053	(0.265)
					T	C					TT	TC	CC		
W10	CON		1,279	0.311	1,381	1,177	0.460				382	617	280		
rs1321666	SCZ	Under 15	57	0.790	63	51	0.447	0.848	(0.848)	0.950 (0.651-1.385)	18	27	12	0.968	(0.968)
		16-25	399	0.484	401	397	0.497	0.068	(0.680)	1.162 (0.991-1.362)	97	207	95	0.096	(0.688)
		26-35	208	0.783	218	198	0.476	0.560	(0.761)	1.066 (0.866-1.311)	58	102	48	0.827	(0.989)
		Over 36	110	0.702	116	104	0.473	0.725	(0.906)	1.052 (0.798-1.386)	29	58	23	0.678	(0.753)
					G	C					GG	GC	CC		
W12	CON		1,280	0.121	2,181	379	0.148				936	309	35		
rs10802003	SCZ	Under 15	57	0.476	102	12	0.105	0.225	(0.563)	0.677 (0.369-1.244)	46	10	1	0.505	(0.670)
		16-25	399	0.423	683	115	0.144	0.819	(0.871)	0.969 (0.773-1.214)	290	103	6	0.344	(0.688)
		26-35	209	0.068	341	77	0.184	0.067	(0.168)	1.299 (0.992-1.703)	135	71	3	**0.010**	(0.080)
		Over 36	110	0.122	189	31	0.141	0.843	(0.928)	0.944 (0.636-1.401)	79	31	0	0.145	(0.364)
					C	T					CC	CT	TT		
W13	CON		1,281	0.292	2,107	455	0.178				872	363	46		
rs10754369	SCZ	Under 15	57	0.592	97	17	0.149	0.530	(0.589)	0.812 (0.480-1.372)	42	13	2	0.700	(0.778)
		16-25	399	0.289	661	137	0.172	0.750	(0.871)	0.960 (0.778-1.184)	277	107	15	0.829	(0.829)
		26-35	209	0.232	324	94	0.225	**0.025**	(0.125)	**1.343 (1.045-1.727)**	122	80	7	**0.016**	(0.080)
		Over 36	110	**0.022**	179	41	0.186	0.714	(0.906)	1.061 (0.744-1.511)	69	41	0	**0.018**	(0.181)
					C	T					CC	CT	TT		
W15	CON		1,280	0.238	2,288	272	0.106				1,018	252	10		
rs3753261	SCZ	Under 15	57	0.577	99	15	0.132	0.357	(0.571)	1.275 (0.730-2.225)	42	15	0	0.508	(0.670)
		16-25	399	0.781	720	78	0.098	0.550	(0.807)	0.911 (0.699-1.188)	325	70	4	0.575	(0.767)
		26-35	209	0.324	370	48	0.115	0.609	(0.761)	1.091 (0.788-1.512)	165	40	4	0.267	(0.381)
		Over 36	110	1.000	187	33	0.150	0.055	(0.386)	**1.484 (1.004-2.194)**	79	29	2	0.080	(0.268)

N: number of subjects, HWE: Hardy-Weinberg equilibrium, MAF: minor allele frequency, FDR: the false discovery rate using the Benjamini-Hochberg procedure, OR: odds ratio, 95% CI: 95% confidence interval, CON: control, SCZ: schizophrenia

ALG1

SNP ID rs number	Affection	Age at onset	N	HWE *P*	Allelic count		MAF	Allelic *P*	(FDR *P*)	OR (95% CI)	Genotypic count			Genotypic *P*	(FDR *P*)
					C	G					CC	GC	GG		
A1	CON		1,281	0.606	1,749	813	0.317				601	547	133		
rs8053916	SCZ	Under 15	57	0.552	77	37	0.325	0.918	(1)	1.034 (0.6924-1.543)	27	23	7	0.835	(1)
		16-25	399	0.816	551	247	0.310	0.695	(0.766)	0.964 (0.812-1.145)	189	173	37	0.833	(0.833)
		26-35	209	0.203	284	134	0.321	0.910	(1)	1.015 (0.813-1.267)	92	100	17	0.336	(0.560)
		Over 36	109	0.608	166	52	0.239	**0.018**	(0.180)	**0.674 (0.488-0.930)**	64	38	7	0.057	(0.285)
					C	T					CC	CT	TT		
A2	CON		1,281	0.825	1,909	653	0.255				713	483	85		
rs9924614	SCZ	Under 15	57	0.739	85	29	0.254	1.000	(1)	0.997 (0.6483-1.534)	32	21	4	0.953	(1)
		16-25	185	0.074	606	192	0.241	0.427	(0.610)	0.926 (0.770-1.115)	23	132	30	0.230	(0.405)
		26-35	209	1.000	305	113	0.270	0.507	(1)	1.083 (0.858-1.368)	111	83	15	0.754	(0.838)
		Over 36	110	0.433	167	53	0.241	0.687	(0.951)	0.928 (0.673-1.280)	65	37	8	0.664	(0.737)
					C	T					CC	TC	TT		
A3	CON		1,279	0.573	2,094	464	0.181				860	374	45		
rs9932909	SCZ	Under 15	57	0.592	97	17	0.149	0.455	(1)	0.791 (0.4679-1.337)	42	13	2	0.586	(1)
		16-25	396	0.702	669	123	0.155	0.097	(0.416)	0.830 (0.668-1.031)	281	107	8	0.209	(0.405)
		26-35	206	0.269	331	81	0.197	0.451	(1)	1.104 (0.849-1.437)	130	71	5	0.293	(0.560)
		Over 36	110	1.000	179	41	0.186	0.856	(0.951)	1.034 (0.726-1.473)	73	33	4	0.946	(0.946)
					C	T					CC	TC	TT		
A4	CON		1,281	1.000	1,977	585	0.228				763	451	67		
rs3760030	SCZ	Under 15	57	1.000	90	24	0.211	0.733	(1)	0.901 (0.5691-1.427)	35	20	2	0.973	(1)
		16-25	399	0.383	622	176	0.221	0.663	(0.766)	0.956 (0.790-1.158)	239	144	16	0.632	(0.790)
		26-35	209	**0.006**	321	97	0.232	0.851	(1)	1.021 (0.799-1.305)	116	89	4	**0.022**	(0.220)
		Over 36	110	0.556	176	44	0.200	0.357	(0.892)	0.845 (0.600-1.191)	69	38	3	0.552	(0.737)
					C	T					CC	TC	TT		
A5	CON		1,277	1.000	2,154	400	0.157				908	338	31		
rs3760029	SCZ	Under 15	57	0.124	96	18	0.158	1.000	(1)	1.010 (0.6035-1.689)	42	12	3	0.228	(1)
		16-25	398	1.000	685	111	0.139	0.259	(0.432)	0.873 (0.695-1.095)	294	97	7	0.539	(0.770)
		26-35	208	0.574	357	59	0.142	0.465	(1)	0.890 (0.662-1.196)	154	49	5	0.682	(0.838)
		Over 36	110	0.121	188	32	0.145	0.771	(0.951)	0.917 (0.621-1.353)	78	32	0	0.250	(0.691)
					T	C					TT	CT	CC		
A6	CON		1,267	0.475	2,187	347	0.137				940	307	20		
rs3760027	SCZ	Under 15	56	0.482	100	12	0.107	0.479	(1)	0.759 (0.4127-1.396)	45	10	1	0.418	(1)
		16-25	396	1.000	688	104	0.131	0.766	(0.766)	0.956 (0.756-1.210)	299	90	7	0.757	(0.833)
		26-35	209	0.052	347	71	0.170	0.081	(0.810)	1.294 (0.979-1.712)	140	67	2	0.060	(0.300)
		Over 36	110	1.000	196	24	0.109	0.303	(0.892)	0.772 (0.498-1.197)	87	22	1	0.630	(0.737)
					G	C					GG	CG	CC		
A7	CON		1,278	0.517	1,564	992	0.388				484	596	198		
rs8045294	SCZ	Under 15	57	0.777	71	43	0.377	0.845	(1)	0.955 (0.6484-1.406)	21	29	7	0.782	(1)
		16-25	398	0.447	508	288	0.362	0.195	(0.416)	0.894 (0.758-1.054)	158	192	48	0.241	(0.405)
		26-35	208	0.078	257	159	0.382	0.828	(1)	0.975 (0.788-1.207)	73	111	24	0.150	(0.375)
		Over 36	110	0.057	145	75	0.341	0.171	(0.855)	0.816 (0.610-1.090)	43	59	8	**0.044**	(0.285)
					C	G					CC	GC	GG		
A8	CON		1,278	0.240	1,289	1,267	0.504				314	661	303		
rs8045473	SCZ	Under 15	57	0.295	55	59	0.518	0.702	(1)	1.091 (0.7497-1.589)	11	33	13	0.626	(1)
		16-25	397	0.547	380	414	0.521	0.208	(0.416)	0.902 (0.769-1.058)	94	192	111	0.227	(0.405)
		26-35	208	0.212	210	206	0.495	1.000	(1)	0.998 (0.811-1.228)	48	114	46	0.735	(0.838)
		Over 36	110	0.085	109	111	0.505	0.833	(0.951)	1.036 (0.787-1.365)	22	65	23	0.345	(0.691)
					C	T					CC	TC	TT		
A9	CON		1,275	0.905	2,204	346	0.136				953	298	24		
rs7195893	SCZ	Under 15	57	1.000	99	15	0.132	1.000	(1)	0.965 (0.5542-1.681)	43	13	1	1.000	(1)
		16-25	398	0.309	707	89	0.112	0.091	(0.416)	0.802 (0.626-1.027)	316	75	7	0.161	(0.405)
		26-35	208	0.764	361	55	0.132	0.938	(1)	0.971 (0.715-1.317)	157	47	4	0.970	(0.970)
		Over 36	110	0.212	190	30	0.136	1.000	(1)	1.006 (0.673-1.503)	80	30	0	0.288	(0.691)
					C	G					CC	CG	GG		
A10	CON		1,281	0.397	2,099	463	0.181				855	389	37		
rs9673733	SCZ	Under 15	57	1.000	93	21	0.184	0.901	(1)	1.024 (0.6308-1.661)	38	17	2	0.858	(1)
		16-25	399	0.706	672	126	0.158	0.150	(0.416)	0.850 (0.685-1.054)	284	104	11	0.243	(0.405)
		26-35	209	0.282	354	64	0.153	0.189	(0.945)	0.820 (0.617-1.089)	152	50	7	0.149	(0.375)
		Over 36	110	0.299	183	37	0.168	0.715	(0.951)	0.917 (0.635-1.323)	74	35	1	0.566	(0.737)

N: number of subjects, HWE: Hardy-Weinberg equilibrium, MAF: minor allele frequency, FDR: the false discovery rate using the Benjamini-Hochberg procedure, OR: odds ratio, 95% CI: 95% confidence interval, CON: control, SCZ: schizophrenia
